# Therapeutic Effects of Noninvasive Electrical Stimulation in Combination Transplantation of Human Adipose‐Derived Stem Cells‐Derived Dopaminergic Neuron on the Monkey Model of Parkinson's Disease

**DOI:** 10.1002/mco2.70595

**Published:** 2026-01-28

**Authors:** Chunhui Huang, Shane Gao, Xiao Zheng, Xichen Song, Jiaxi Wu, Kai Liao, Jiawei Li, Yingqi Lin, Caijuan Li, Yaqun Lu, Jiahao Feng, Huiyi Wei, Lu Wang, Hao Xu, Wei Wang, Yizhi Chen, Jianhao Wu, Jiale Gao, Junzhu Song, Chunxiang Shi, Jun Zhang, Sen Yan

**Affiliations:** ^1^ The Sixth Affiliated Hospital of Jinan University Dongguan China; ^2^ The School of Traditional Chinese Medicine Jinan University Guangzhou China; ^3^ State Key Laboratory of Bioactive Molecules and Druggability Assessment, Guangdong Basic Research Center of Excellence for Natural Bioactive Molecules and Discovery of Innovative Drugs, Guangdong Provincial Key Laboratory of Non‐human Primate Research, Guangdong‐Hong Kong‐Macau Institute of CNS Regeneration Jinan University Guangzhou China; ^4^ Department of neurosurgery Shanghai East Hospital, School of Medicine, Tongji University Shanghai China; ^5^ Stomatological Hospital, School of Stomatology Southern Medical University Guangzhou China; ^6^ Department of Nuclear Medicine and PET/CT‐MRI Center The First Affiliated Hospital of Jinan University Guangzhou China; ^7^ Department of Neurology, Guangzhou Red Cross Hospital, Faculty of Medical Science Jinan University Guangzhou Guangdong China; ^8^ QuanYan Biotechnology Limited Company Shanghai China; ^9^ Department of Traditional Chinese Medicine The Seventh Affiliated Hospital of Sun Yat‐sen University Shenzhen China; ^10^ The Fifth Affiliated Hospital (Heyuan Shenhe People's Hospital) Jinan University Heyuan China; ^11^ Shenzhen LUZE Biological Technology Co., Ltd. Shenzhen China; ^12^ Traditional Chinese Medicine Department The First Affiliated Hospital of Jinan University Guangzhou China

**Keywords:** human adipose‐derived stem cells, neuroinflammation, nonhuman primate, noninvasive electrical stimulation, Parkinson's disease, Serpin family A member 3

## Abstract

Parkinson's disease (PD) is a neurodegenerative disease caused by the loss of dopaminergic neurons (DNs). Currently, there is no treatment that can cure PD. Deep brain stimulation has been used to treat PD due to its good effectiveness, but there are safety issues. Therefore, noninvasive electrical stimulation (NES) may be an effective and safe strategy for the treatment of PD. Here, we performed NES treatment and NES combined with human adipose‐derived stem cells‐induced DN transplantation (NES‐DN) on the PD monkey model to explore the therapeutic effect of NES on PD. The results show that NES or NES‐DN can increase dopamine levels, improve mitochondrial dysfunction, reduce neuroinflammation, enhance synaptic function, and protect TH neurons, thereby improving the movement disorders of PD. Moreover, NES/NES‐DN may exert immunomodulatory effects by regulating serpin family A member 3 in PD monkeys. Our results support the scientific basis and preclinical evidence for NES in the treatment of PD. Not only does NES alone improve PD, but NES combined with stem cell therapy can greatly enhance the therapeutic effect of PD.

## Graphical abstract

Our results showed that noninvasive electrical stimulation (NES) alone or in combination with human adipose‐derived stem cells (hADSCs)‐induced dopaminergic neuron (NES‐DN) improved the behavioral deficits and pathological features of the MPTP‐induced PD monkey model. These findings provide a solid scientific basis and preclinical evidence for NES treatment of PD. It also provides new guidance for the combination of NES with other therapies, especially stem cell transplantation therapy.

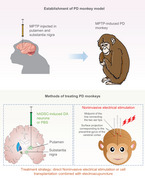

## 1 Introduction

Parkinson's disease (PD) is the second most common neurodegenerative disorder, with major clinical manifestations including bradykinesia, resting tremor, muscle and joint stiffness, and gait imbalance, affecting 2–3% of people aged 65 years and older population [1, 2]. Although both genetic and environmental factors are involved in the development of PD, its cause remains unclear. The development of PD symptoms is mainly caused by the severe loss of dopaminergic neurons in the substantia nigra (SNpc) of the midbrain and subsequent depletion of striatal dopamine [3]. Pharmacological replacement therapies based on dopamine are beneficial for initial motor symptoms; however, their efficacy is limited and they are associated with adverse effects [4]. There are also nondopaminergic approaches to address motor and nonmotor symptoms such as deep brain stimulation (DBS) and electrical stimulation [5]. Experimental therapies to restore striatal dopamine through gene‐based and cell‐based approaches have been reported, and clinical studies of gene therapy and cell therapy are also gradually underway [1]. However, there is still no cure for PD, so it is urgent to explore effective treatments for PD.

DBS is a neuromodulatory treatment used to manage symptoms of PD and other neurological and psychiatric disorders [5]. Electrodes are implanted chronically in disease‐related brain regions, and pulsatile electrical stimulation is delivered with the goal of restoring neural circuit function. Although it has been shown to have good efficacy, long‐term brain implants are difficult to guarantee safety [6]. Noninvasive electrical stimulation (NES) has been gradually proposed for the treatment of PD, but its related research is very limited. For example, repetitive transcranial magnetic stimulation (rTMS) and transcranial direct current stimulation (tDCS) are promising noninvasive cortical stimulation methods for the adjunctive treatment of PD movement disorders [7]. In other studies, rTMS and tDCS have made important contributions to mood enhancement in PD [8, 9]. In addition, electroacupuncture (EA), as part of complementary medicine, has also been explored in the treatment of PD [10, 11]. In PD models, EA treatment can increase the release of various neuroprotective agents and slow down the cell death process and is therefore considered a neuroprotective therapy [12]. In addition, in clinical studies, EA treatment at different acupoints can activate survival pathways, regulate neurotransmitters, and increase neurotrophic factors to improve the survival rate of dopaminergic neurons, thereby benefiting PD patients [13, 14]. Although NES therapy for PD is promising, the related efficacy research and mechanism exploration are very limited and deserve further exploration.

Moreover, stem cells have shown great potential in treating PD. Mesenchymal stem cells (MSCs) provide a simple cell source with low rates of immune rejection and tumorigenesis and almost no adverse effects and treatment‐related complications [15]. The results of several PD animal models have shown that MSCs‐derived secretosomes have great benefits in treating PD, including regenerative and protective effects on DA neurons [16]. Combining EA with stem cell transplantation is considered a more promising strategy to enhance the therapeutic effects of stem cells [17]. It has been reported that EA can promote the release of anti‐inflammatory factors from MSCs in the central nervous system (CNS) in mice [18]. Furthermore, new research shows that cotreatment of electroconvulsive therapy (ECT) and MSCs transplantation synergistically improves symptoms in 1‐methyl‐4‐phenyl‐1,2,3,6‐tetrahydropyridine (MPTP) mouse models by increasing dopamine levels and reducing proinflammatory cytokines [19]. Therefore, NES combined with stem cells or cell transplantation can be considered as a highly promising therapeutic strategy, which requires more research and verification.

In order to verify the effect of NES and the combination of NES and MSCs in treating PD and find its possible mechanism, we injected neurotoxicant MPTP into the striatum and SNpc of nonhuman primates (NHPs) to generate a PD monkey model [20, 21]. We then induced human adipose‐derived stem cells (hADSCs) to differentiate into DA neurons (DN) in vitro, and treated PD monkeys with NES or NES combined with hADSCs‐induced DA neuron transplantation (NES‐DN). Our findings indicate that both NES and NES‐DN can improve behavioral phenotypes and pathology in MPTP monkeys by increasing brain DA levels and neurotransmitter transmission, improving mitochondrial dysfunction, as well as inhibiting neuroinflammation. It is worth mentioning that the combined treatment effect of NES and hADSCs‐induced DA neurons is better than that of NES alone, which provides new insights into the treatment of PD by NES and contributes to the development and application of NES in combination with other therapies.

## 2 Results

### 2.1 NES and NES‐DN Rescue Motor Deficits in Parkinsonian Monkeys

**FIGURE 1:**
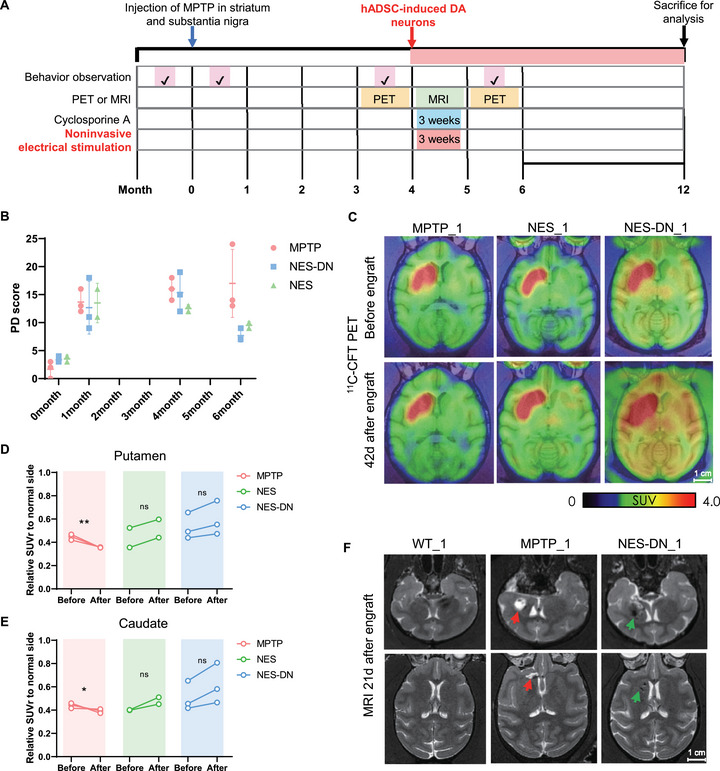
NES and NES‐DN can improve the behavioral disorders and DAT levels in PD monkeys. (A) Schematic diagram of MPTP monkey model modeling method and treatment strategy. (B) Motor behavior scores before and after treatment. MPTP (*n* = 3), NES (*n* = 2), and NES‐DN (*n* = 3). (C) Display the levels of DAT in the brain of MPTP monkeys before and after treatment using PET images of 11C‐CFT tracer. (D and E) Quantitative analysis of the binding strength of 11C‐CFT PET to putamen (D) and caudate (E) in MPTP monkeys. MPTP (*n *= 3), NES (*n* = 2), and NES‐DN (*n* = 3). (F) MRI detection of transplantation of hADSCs induced DA nerve grafts labeled with iron oxide nanoparticles.

The unique motor skills and neuroanatomical complexity of NHPs share similarities with humans, providing a bridge to understand the pathophysiology of PD and an accurate model [22]. The NHPs model treated with MPTP is an important preclinical model of PD, which can simulate the movement disorders and pathological manifestations of PD and is beneficial to evaluate the safety and effectiveness of novel treatment strategies [20, 23]. In order to explore the value of NES and NES combined with NES‐DN therapy for PD, we designed an experimental process for the establishment and treatment of the MPTP monkey model (Figure 1A). The behavior of the monkeys was assigned scores before and after the modeling, and it was found that the monkeys had significant behavioral disorders after MPTP injection (Figure 1B).

We then aspirated adipose tissue from healthy patients, isolated, and cultured it to obtain hADSCs, which were used to differentiate into DA neurons combined with NES treatment [24]. To differentiate hADSCs into mesodermal trilineage cells and dopamine neurons, we incubated P3‐stage hADSCs in adipogenic/osteogenic/chondrogenic medium according to established methods [25, 26]. The results showed that hADSCs derived from adipose tissue highly expressed the MSC markers CD44, CD73, CD 90, and CD105 (Figure S1A,B). Furthermore, these hADSCs can be conditionally induced into trileage mesodermal cells of adipocyte, osteoblast and chondrocyte (Figure S1C,D). Importantly, hADSCs can be gradually induced to differentiate into dopaminergic neuron‐like (TH‐positive) cells (Figure S1E,F), which can secrete dopamine. To better detect the presence of cells, we labeled hADSCs with EGFP lentivirus before transplantation and differentiated them into dopaminergic neuron‐like cells (Figure S1G).

To ensure that there was no spontaneous recovery of symptoms in PD monkeys, we scored the behavior of MPTP monkeys again before cell transplantation and found that their performance did not improve over time (Figure 1B and Movie S1). MPTP was unilaterally injected into the right hemisphere of the monkey, which would cause contralateral (left) motor dysfunction due to the crossed nigrostriatal pathways. For example, WT monkeys can easily grab food with their left and right hands. The PD monkeys were injected in the right hemisphere, and it can be seen that the left hand has obstacles and cannot perform the grasping task normally, so the monkey usually uses the uninjured right hand to grasp. At the same time, we used DAT tracer to conduct PET detection on PD monkeys, and the results showed that the DAT signal on the MPTP‐treated side almost completely disappeared (Figure 1C–E). The above results prove that the MPTP‐induced monkey model is relatively stable. MPTP monkeys were randomly divided into three groups (Table 1), including MPTP group, NES group, and NES‐DN group. Before transplantation, iron oxide nanoparticles were added to the differentiated DA cells to facilitate magnetic resonance imaging (MRI) detection of cell transplantation. Subsequently, differentiated DA neurons were transplanted into the SNpc and striatum on the model side of the NES‐DN group, and the immunosuppressant Cyclosporine A was administered for 3 consecutive weeks. The MPTP group and NES group were injected with the same amount of 1×PBS. Furthermore, the NES group and NES‐DN group were given NES treatment for 3 weeks to explore the efficacy of NES or NES combined with hADSCs‐induced DA neurons.

**TABLE 1:** Establishment of MPTP monkey model and information on treatment methods.

Number	80 µg/µL MPTP in right side	Treatment	Number of engrafted cells (×10^6^ counts)
NES‐DN_1	20 µL in stritum; 10 µL in substantia nigra	DN in stritum and substantia nigra, and NES	5 × 10^6^ in stritum, 2 × 10^6^ in substantia nigra
NES‐DN_2	20 µL in stritum; 10 µL in substantia nigra	DN in stritum and substantia nigra, and NES	5 × 10^6^ in stritum, 2 × 10^6^ in substantia nigra
NES‐DN_3	20 µL in stritum; 10 µL in substantia nigra	DN in stritum and substantia nigra, and NES	5 × 10^6^ in stritum, 2 × 10^6^ in substantia nigra
NES_1	20 µL in stritum; 10 µL in substantia nigra	PBS in stritum and substantia nigra, and NES	Same volume of PBS
NES_2	20 µL in stritum; 10 µL in substantia nigra	PBS in stritum and substantia nigra, and NES	Same volume of PBS
MPTP_1	20 µL in stritum; 10 µL in substantia nigra	PBS in stritum and substantia nigra	Same volume of PBS
MPTP_2	20 µL in stritum; 10 µL in substantia nigra	PBS in stritum and substantia nigra	Same volume of PBS
MPTP_3	20 µL in stritum; 10 µL in substantia nigra	PBS in stritum and substantia nigra	Same volume of PBS
WT_1	Same volume of PBS in stritum and substantia nigra	PBS in stritum and substantia nigra	Same volume of PBS
WT_2	Same volume of PBS in stritum and substantia nigra	PBS in stritum and substantia nigra	Same volume of PBS
WT_3	Same volume of PBS in stritum and substantia nigra	PBS in stritum and substantia nigra	Same volume of PBS

Twenty‐one days after transplantation, MRI results showed that there was a focus signal in the MPTP monkeys without transplanted cells, while the signal of iron oxide nanoparticles could be seen in the monkeys in the NES‐DN group with transplants (Figure 1F). After 6 weeks of treatment, PET results showed that both NES and NES‐DN treatments partially restored the DAT signal loss caused by MPTP, while the DAT signal loss in monkeys in the MPTP group was aggravated (Figure 1C–E). Moreover, after 2 months of treatment, the PD scores of both NES and NES‐DN were reduced (Figure 1B). Interestingly, the loss of flexibility of left hand caused by MPTP injection was improved after NES and NES‐DN treatments, leading to successful grasping of food (Movie S2 and Movie S3). Specifically, before treatment, when food was given to the affected left hand, the PD monkey would still use the uninjured right hand to obtain food (Movie S1, Movie S2 before treatment and Movie S3 before treatment). Importantly, after treatment, the food was given to the monkey's damaged left hand, and it could grasp and eat the food, indicating that the monkey's behavioral disorder was restored after treatment (Movie S2 after treatment and Movie S3 after treatment), even though it still used its right hand most of the time. The above studies have shown that hADSCs‐induced DA neurons were successfully transplanted, and both NES and NES‐DN treatment improved behavioral impairment and DAT loss in PD monkeys.

### 2.2 NES and NES Combined With hADSCs‐Induced DA Neuron Grafts Protect TH‐Positive Neurons and Increase Dopamine Levels

**FIGURE 2:**
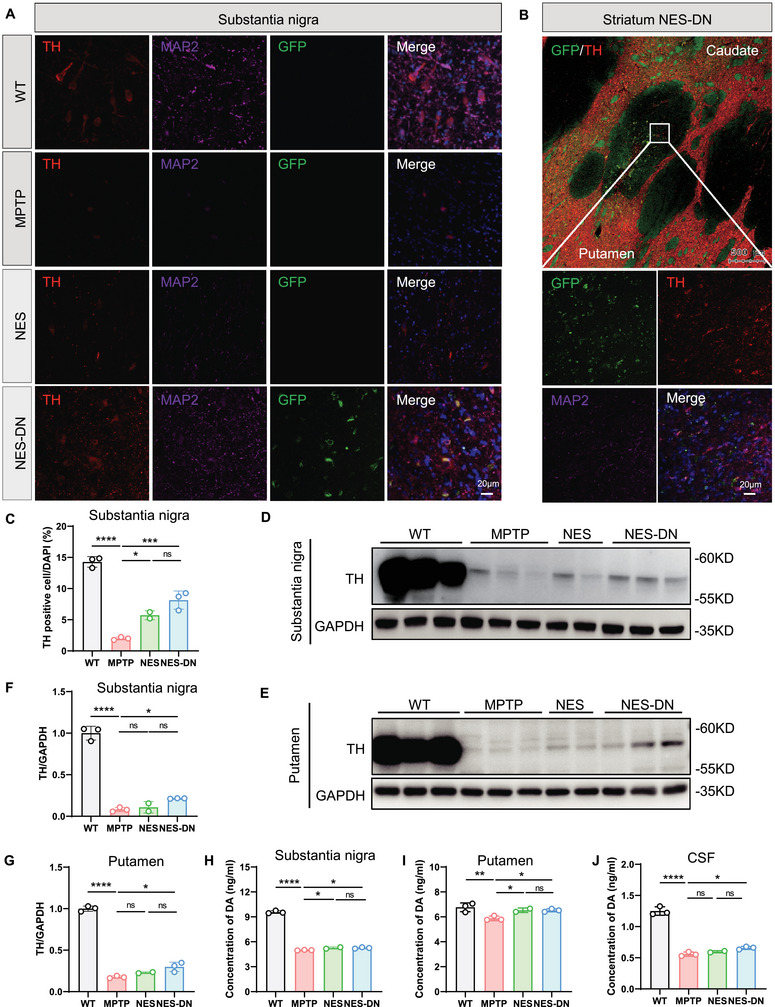
NES and NES‐DN can protect TH positive neurons and increase dopamine levels. (A and B) Representative images of immunofluorescence staining show the colabeling of TH (red), MAP2 (purple), and GFP (green) in the substantia nigra (A) and striatum (B) to demonstrate the survival of transplanted cells, scale bar = 20 µm. (C) Quantitative statistics of the number of TH positive neurons in the substantia nigra. WT (*n* = 3), MPTP (*n* = 3), NES (*n* = 2), and NES‐DN (*n* = 3), **p < 0.01, ***p < 0.005, ***p < 0.001. (D and E) Western blot analysis of TH protein expression levels in the substantia nigra (D) and putamen (E). (F and G) Quantitative analysis of TH protein expression levels in substantia nigra (F) and putamen (G). WT (*n* = 3), MPTP (*n* = 3), NES (*n* = 2), and NES‐DN (*n* = 3), *p < 0.05, ****p < 0.001. (H, I, and J) Quantitative statistics of DA levels in the substantia nigra (H), putamen (I), and CSF (J) detected by ELISA. WT (*n* = 3), MPTP (*n* = 3), NES (*n* = 2), and NES‐DN (*n* = 3), *p < 0.05, **p < 0.01, ****p < 0.001.

In order to further prove the efficacy of NES and NES‐DN, we collected monkey brain tissue for physiological and biochemical analysis. Through immunofluorescence staining, we found that DA neurons transplanted into the SNpc and striatum survived well and expressed GFP^+^, TH^+^, and MAP2^+^ signals (Figure 2A–C). Moreover, western blots results showed that after treatment with NES and NES‐DN, the reduction in TH caused by MPTP treatment was restored, and the recovery of NES‐DN was better than that of NES alone (Figure 2D–G). Furthermore, we analyzed dopamine levels in the SNpc, putamen, and cerebrospinal fluid (CSF), and the results showed that both NES and NES‐DN treatments increased dopamine levels in SNpc, putamen, and CSF (Figure 2H–J). Therefore, NES and NES‐DN treatment may protect DA neurons and produce therapeutic effects on PD.

### 2.3 NES and NES‐DN Treatment Alleviate Gene Expression Dysregulation in Putamen of MPTP Monkeys

**FIGURE 3:**
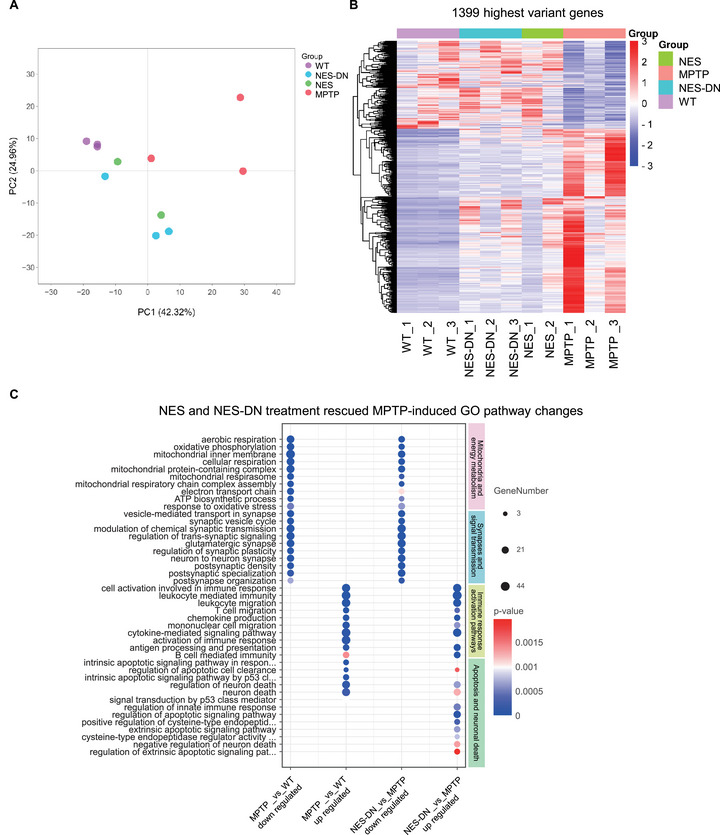
RNA seq reveals the improvement of NES and NES‐DN treatment on MPTP monkeys. (A) PCA analysis of similarities between WT, MPTP, NES, and NES‐DN. (B) Heat map analysis shows the gene expression of WT, MPTP, NES, and NES‐DN. (C) Bubble chart showing significantly changed paths in GO enriched results.

Although NES has been widely reported to be helpful in the treatment of PD, the mechanism of NES treating PD has been rarely studied [27, 28, 29, 30]. To explore the potential mechanism of action of NES and its synergistic effect with transplanted cells, we performed RNA seq analysis on monkey putamen. In principal component analysis (PCA), different groups can be clustered together separately. In particular, the MPTP group is further away from the WT group, while the NES and NES‐DN groups are closer to the WT (Figure 3A). In addition, we performed cluster analysis on the sequencing results of the samples, and the results showed that as many as 1399 differential genes were generated after MPTP treatment. Importantly, the changes in these differential genes were alleviated after treatment with NES and NES‐DN, gradually tending to the gene expression levels of WT (Figure 3B). GO enrichment analysis of these differentially expressed genes showed that many pathways were altered in the MPTP group compared with WT, including downregulation of mitochondria‐related pathways, synaptic and signaling pathways, and upregulation of immune response activation pathways, while NES‐DN treatment significantly improve the imbalance of these pathways, apoptosis, and neurons death pathways (Figure 3C). These results demonstrate that treatment with NES and NES‐DN can alleviate MPTP‐induced transcriptional dysregulation in PD.

**FIGURE 4:**
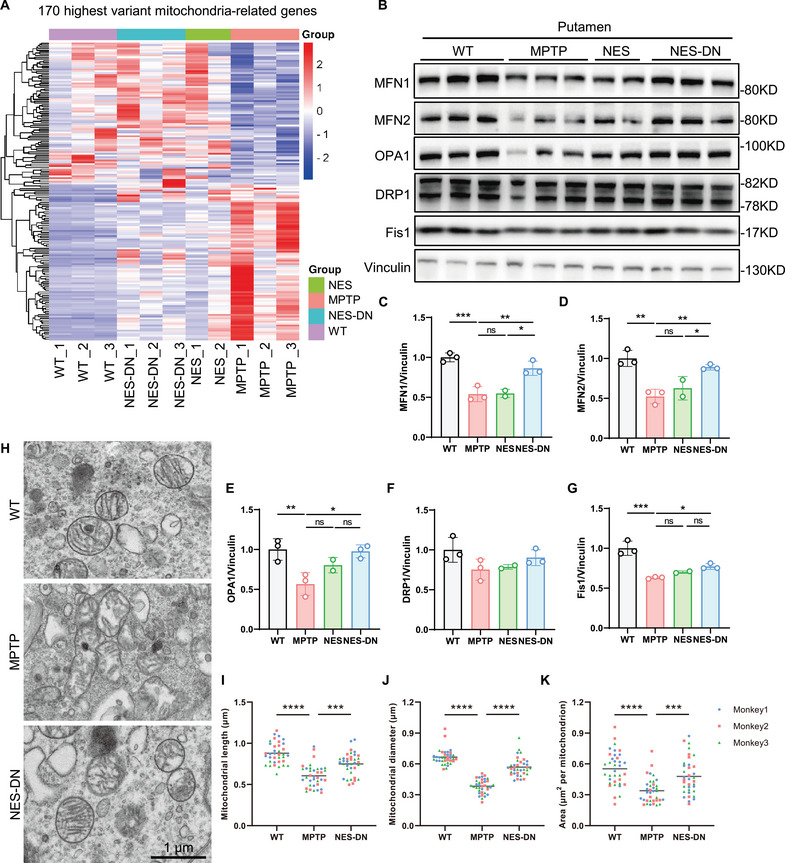
Improved expression of mitochondrial related proteins after NES and NES‐DN treatment. (A) Heat maps of mitochondrial related gene changes in WT, MPTP, NES, and NES‐DN. (B) Western blot images of mitochondrial related proteins. (C–G) Quantitative statistical results of changes in mitochondrial related protein levels, MFN1 (C), MFN2 (D), OPA1 (E), DRP1 (F), Fis1 (G). WT (*n* = 3), MPTP (*n* = 3), NES (*n* = 2), and NES‐DN (*n *= 3), *p < 0.05, **p < 0.01, ***p < 0.005. (H) Representative images of mitochondria of WT, MPTP, and NES‐DN in putamen, scale bar = 1 µm. (I–K) Quantitative statistics of mitochondria of WT, MPTP, and NES‐DN in putamen, including mitochondrial length (I), mitochondrial diameter (J), and mitochondrial area (K). WT (*n* = 3), MPTP (*n* = 3), and NES‐DN (*n* = 3), ***p < 0.005, ****p < 0.001.

A number of studies suggest that mitochondrial dysfunction may play a key role in DA neuron loss [31, 32]. The direct link between mitochondrial dysfunction and PD comes from the discovery of PD symptoms caused by MPTP abuse [33]. Therefore, we first focused on whether there were abnormalities in mitochondria‐related markers. From the RNA seq results, it can be found that mitochondria‐related genes were significantly changed after MPTP treatment, and NES and NES‐DN treatments partially restored the dysregulation of these genes (Figure 4A). Further western blots results confirmed that the expression levels of key proteins in mitochondrial fusion and fission such as MFN1, MFN2, OPA1, and Fis1 were reduced with MPTP treatment. However, these markers were significantly restored after NES‐DN treatment (Figure 4B–G). Through transmission electron microscopy examination, we also found that MPTP‐treated shell and nucleus mitochondria were damaged, including shortened mitochondrial length and diameter, reduced size, and dissolution of the mitochondrial inner membrane, accompanied by the disappearance of mitochondrial cristae, while the damage was improved after NES‐DN treatment (Figure 4H–K). Therefore, NES‐DN treatment has a favorable effect on MPTP‐induced mitochondrial dysfunction.

### 2.4 NES and NES‐DN Treatment Reduce Neuronal and Synaptic Damage

**FIGURE 5:**
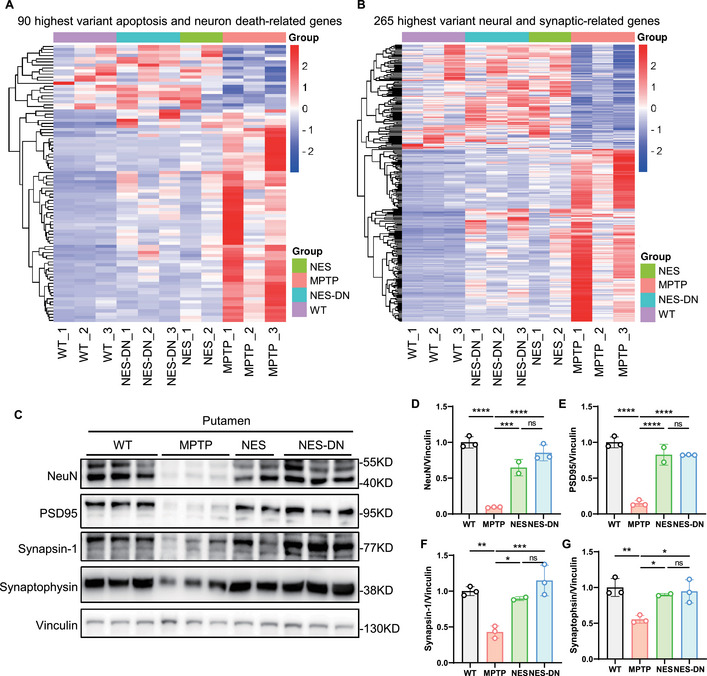
The damage to neurons and synapses is reduced after NES and NES‐DN treatment. (A) Heat maps showing changes in apoptosis and neuron death related genes in WT, MPTP, NES, and NES‐DN. (B) Heat maps showing changes in neural and synaptic related genes in WT, MPTP, NES, and NES‐DN. C, Western blot results of protein levels related to neurons and synapses. (D–G) Quantitative statistical results of changes in neuronal and synaptic related protein levels, NeuN (D), PSD95 (E), synapsin‐1 (F), synaptophysin (G). WT (*n* = 3), MPTP (*n* = 3), NES (*n* = 2), and NES‐DN (*n* = 3), *p < 0.05, **p < 0.01, ***p < 0.005, ****p < 0.001.

NES treatment is thought to modulate synaptic function and neurotransmitter release [12, 34, 35]. MPTP treatment led to downregulation of gene expression levels of synapse‐related signaling pathways and upregulation of gene expression associated with neuronal death pathways (Figure 5A,B). Further, the clustering results showed that apoptosis and neuronal death, as well as neuronal and synapse‐related genes were significantly altered, and NES/NES‐DN treatment rescued this change. Western blots experiments verified that the reduction of NeuN, PSD95, synapsin‐1, and synaptophysin, proteins related to neurons and synapses, caused by MPTP treatment, was significantly restored after NES and NES‐DN treatment (Figure 5C–G). These results demonstrate that NES and NES‐DN treatment can effectively increase synaptic function and neurotransmitter transmission.

### 2.5 NES and NES‐DN Treatment Improved Neuroinflammation in MPTP Monkeys

Neurohistological and neuroimaging evidence supports the existence of ongoing and terminal neuroinflammatory processes in PD [36]. Alterations in inflammatory markers and immune cell populations in PD may trigger or exacerbate neuroinflammation and perpetuate neurodegenerative processes. In mouse models, NES treatment can regulate PD‐related symptoms through immune regulation, and immune regulation is considered an important means of PD treatment [37, 38, 39]. However, its immunomodulatory effect in PD patients is unknown. In addition to this, MSCs are believed to have powerful immunosuppressive effects. Thus, we focused on the effects of NES and NES‐DN on MPTP‐induced neuroinflammation.

**FIGURE 6:**
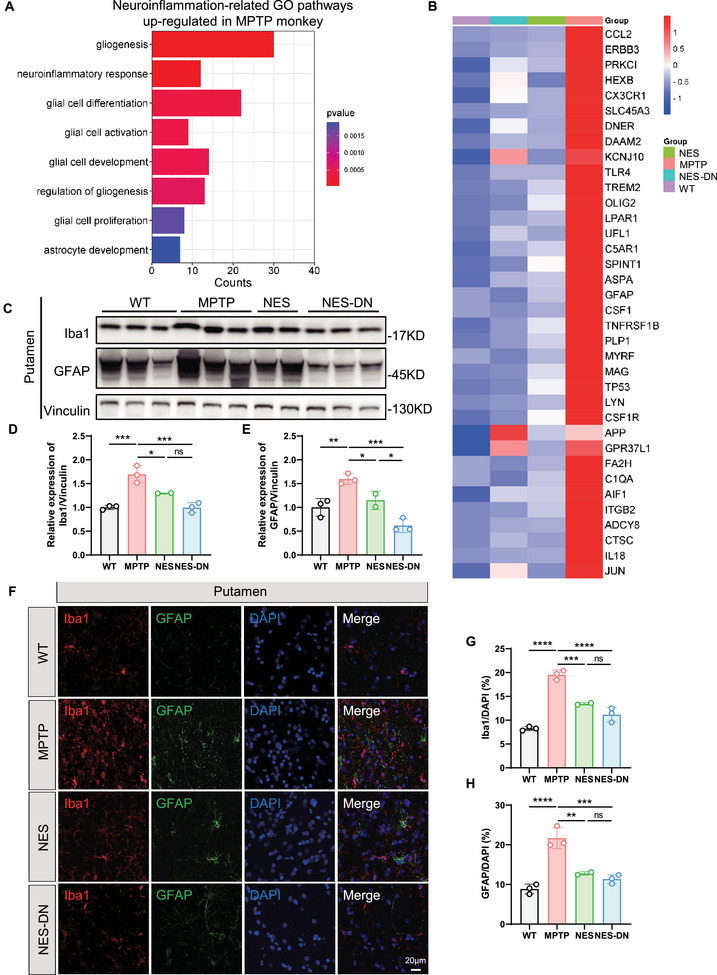
NES and NES‐DN treatments regulated the level of neuroinflammation in putamen. (A) Enrichment results of significantly upregulated GO pathways related to neuroinflammation in the MPTP monkey model. (B) Heat map of changes in significantly upregulated neuroinflammatory genes in the MPTP monkey model after treatment with EA and NES‐DN. (C) Western blot results of Iba1 and GFAP in putamen. (D and E) Quantitative statistical results of changes in Iba1 (D) and GFAP (E) protein levels. WT (*n* = 3), MPTP (*n* = 3), NES (*n* = 2), and NES‐DN (*n* = 3), *p < 0.05, **p < 0.01, ***p < 0.005. (F) Representative images of Iba1 and GFAP immunofluorescence staining in putamen, scale bar = 20 µm. (G and H) Quantitative statistics of Iba1 (G) and GFAP (H) positive cells. WT (*n* = 3), MPTP (*n* = 3), NES (*n* = 2), and NES‐DN (*n* = 3), **p < 0.01, ***p < 0.005, ****p < 0.001.

Since GO enrichment analysis has shown that immune pathways are significantly upregulated in MPTP in putamen, NES‐DN treatment alleviates the activation of immune pathways (Figure 3C). More specifically, neuroinflammation‐related pathways such as gliosis, glial cell activation, and glial cell differentiation were significantly activated in MPTP (Figure 6A). The genes enriched in these activated pathways were all significantly upregulated in MPTP‐treated monkeys, while both NES and NES‐DN treatment significantly reduced the expression of these genes (Figure 6B). Through western blots experiments, we found that the expression of Iba1 and GFAP was indeed increased in putamen of MPTP monkeys, while NES and NES‐DN treatment reduced the levels of these proteins (Figure 6C–E). Immunofluorescence staining also demonstrated that Iba1 and GFAP‐positive cells increased significantly in MPTP monkeys, while glial cell proliferation was significantly reduced to varying degrees in both NES and NES‐DN treatment groups (Figures 6F–H and S2). Therefore, NES and NES‐DN have strong regulatory effects on neuroinflammation in MPTP monkeys.

### 2.6 Serpin Family A Member 3 may be a Key Target of PD Induced Neuroinflammation

**FIGURE 7:**
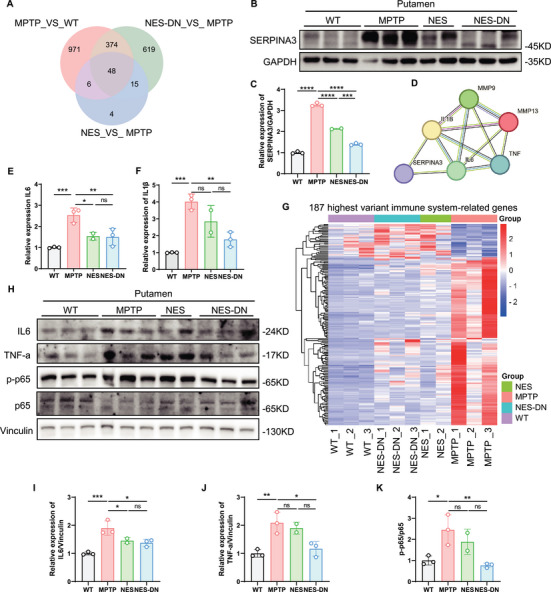
NES and NES‐DN attenuate the immune response by lowering SERPINA3. (A) RNA seq analysis revealed changes in 48 genes coregulated by NES and NES‐DN treatments, as shown in the Venn diagram. (B) Western blot results of SERPINA3 in putamen. C, Quantitative statistical results of changes in SERPINA3 protein levels. WT (*n* = 3), MPTP (*n* = 3), NES (*n* = 2), and NES‐DN (*n* = 3), ****p < 0.001. (D) STRING analysis shows that SERPINA3 can regulate IL‐6 and IL‐1β. (E and F) QPCR results display IL‐6 (E) and IL‐1β (F) RNA quantification statistical chart. (G) RNA seq analysis reveals a heatmap of immune related gene changes in MPTP monkeys after treatment. (H) Western blot results of immune related proteins in putamen. (I–K) Quantitative statistical results of changes in immune related protein levels of IL6 (I), TNF‐α (J), p‐p65/p65 (K). WT (*n* = 3), MPTP (*n* = 3), NES (*n* = 2), and NES‐DN (*n* = 3), *p < 0.05, **p < 0.01, ***p < 0.005.

To identify key factor that might modulate neuroinflammation in MPTP monkeys, we intersected genes that were significantly altered in MPTP and genes that were significantly reverted by both treatments (Figure 7A). The Venn diagram showed that 48 genes may have key regulatory roles in MPTP monkeys. Heatmap analysis of these genes revealed that the expression levels of these 48 DEGs were significantly altered in the MPTP group, while after treatment they became more similar to those in the WT group (Figure S3A). Among them, we found that an important inflammation‐related gene, SERPINA3 (Serpin Family A Member 3), was highly expressed in MPTP, and NES and NES‐DN treatment restored its expression (Figure S3B–F). Western blots verified that highly expressed SERPINA3 in MPTP decreased significantly after treatment (Figure 7B,C). Previous studies have demonstrated that SERPINA3, as an immune related protein, is a biomarker for various diseases [40, 41, 42]. In the CNS, the expression of SERPINA3 derived from astrocytes is influenced by IL‐1β, TNF‐a, and IL‐6, and inhibiting SERPINA3 can improve IL‐1β‐induced inflammation [43, 44, 45, 46]. Similarly, we also validated in human microglia (HMC3) that treatment with MPP^+^ can generate inflammatory activation consistent with LPS, including an increase in mRNA levels of IL‐6 and IL‐1β expression (Figure S4A–D). However, LPS treatment did not lead to an increase in SERPINA3, while MPP^+^ treatment resulted in a significant increase in SERPINA3 (Figure S4B). Moreover, overexpression of SERPINA3 in HMC3 can significantly increase the expression levels of IL‐6 and IL‐1β, similar to LPS (Figure S4E–G). The above results indicate that MPP^+^‐induced inflammation may be mediated by increased expression levels of SERPINA3, and inhibition of SERPINA3 may have a regulatory effect on similar PD neuroinflammation.

Querying the STRING database, we found that SERPINA3 has regulatory effects on IL‐1β and IL‐6 (Figure 7D). QPCR detection results confirmed that IL‐1β and IL‐6 were increased in MPTP monkey putamen and decreased after treatment with NES and NES‐DN (Figure 7E,F). In addition, we found that in addition to neuroinflammation, 187 immune‐related genes were dysregulated in the MPTP group, which gradually returned to WT levels after treatment (Figure 7G). We selected important immune factors such as IL‐6 and TNF‐a, as well as the immune regulatory factor p65 for detection, and the results showed that these immune factors that were significantly upregulated in MPTP were significantly restored by NES‐DN, such as the decrease in IL‐6, TNF‐a, and p‐p65/p65 (Figure 7H–K). The above studies indicate that SERPINA3 may be a key immune regulatory factor for NES and NES‐DN in the treatment of PD.

## 3 Discussion

In this study, we used NHPs to study the efficacy and mechanism of NES in PD models for the first time, proving that NES can produce DA neuron protection by improving mitochondria, enhancing synaptic function, and reducing neuroinflammation. More importantly, our combination of NES and hADSCs‐induced DA neuron has a better therapeutic effect on PD than NES alone, which also provides important scientific basis for NES to assist other therapies, especially cell/stem cell transplantation therapy.

Electrical current has been shown to activate and restore connections between damaged neurons [47, 48]. DBS, an effective treatment for PD, is an invasive neurosurgery. As a pacemaker for the brain, DBS can alleviate some of the movement disorder features of PD patients [49]. Recently, researchers reported that by placing an epidural electrical stimulation (EES) device in the lumbosacral spinal cord, researchers can precisely stimulate the motor neurons that control the lower limbs in space and time, thereby alleviating movement disorders in patients with advanced PD [50]. However, DBS and EES require implanted electrodes, which pose safety risks and the stimulation target cannot be changed, which has certain limitations. In contrast, in addition to its good efficacy, NES has advantages in terms of safety and convenience, as well as being minimally invasive or even noninvasive. NES only requires electrical stimulation of specific brain areas to work. This study demonstrated that NES stimulation of specific scalp areas (The midpoint of the line connecting the two ear tips and the surface projection corresponding to the precentral gyrus of the cerebral cortex are also considered.) in PD monkeys can produce significant therapeutic effects. Our results indicate that NES/NES‐DN not only protects DA neurons from damage and increases DA levels in the brain, but also improves behavioral deficits in PD (Figure 1).

Since DA neuron loss in PD patients is irreversible, the beneficial effect of treatment with NES alone is limited. Restoring DA neurons in the brains of PD patients may be the most direct method, including induced pluripotent stem cells (iPSCs), MSCs, NSCs, and embryonic stem cells (ESCs) [51]. There are currently close to 60 clinical studies involving stem cell therapy for PD registered on the clinicaltrails.gov website, and there are also endless preclinical studies on stem cell therapy for PD. For example, researchers transplanted DA neurons derived from human parthenogenetic stem cells into the brains of PD monkeys and observed the survival of the implanted cells and significant improvement of PD symptoms [52]. Another study transplanted dopaminergic progenitor cells differentiated from HLA‐matched iPSCs into the putamen of a MPTP‐induced PD monkey model and improved symptoms, demonstrating the effectiveness and safety of iPSCs transplantation [53]. Unlike iPSCs that induce differentiation into multiple DA neurons and play a cell replacement role, the efficacy of MSCs may mainly rely on their secretory properties rather than their differentiation properties. In addition to the need to consider differentiation efficiency, MSCs have the advantages of wide sources, easy extraction, low immunogenicity and no ethical controversy [54]. It is worth mentioning that current clinical research on PD treated with MSCs has received positive reports (https://www.biospace.com/article/releases/fda‐authorizes‐novel‐stem‐cell‐trial‐for‐parkinson‐s/). Therefore, we chose hADSCs‐induced DA neurons to combine with NES to explore the synergistic effect of NES and MSCs.

Overall, both NES and NES‐DN produced beneficial effects on the MPTP‐induced monkey model, including improvement of behavioral disorders and increase in dopamine levels. Although a direct comparison between NES and NES‐DN may not be reasonable due to the limited sample size of the NES group, NES‐DN seems to have a better therapeutic effect based on the sample individuals. Compared with NES treatment alone, the advantages of combined treatment (NES‐DN) are outstanding, including improvements in synapses and mitochondria, especially neuroinflammation. Considering that NES and MSCs themselves have powerful modulatory effects on synaptic function and immune regulation, we speculate that the combination of NES and MSCs may strengthen these effects and thus have significant beneficial effects on the symptoms of PD [18, 55]. This suggests that the combined use of NES with other therapies may produce better efficacy. For example, although stem cell and acupuncture therapy have different treatment mechanisms, they both have the advantage of multiple targets, and both can better promote the recovery of ischemic stroke to a certain extent [56, 57]. In another study, NES, as an adjuvant therapy for MSCs‐derived motor neuron transplantation, accelerated neural network reconstruction and recovery of spinal cord function after spinal cord injury by increasing local production of NT‐3 [58]. In addition, studies have shown that the coapplication of ECT and MSCs transplantation produces a synergistic effect through the increase of DA and the decrease of proinflammatory cytokines, thereby improving the movement disorders of MPTP mice [19]. Considering the simplicity and noninvasiveness of NES, NES combined with other therapies can be a valuable strategy for the treatment of PD, such as the combination of NES and iPSCs or the combination of NES and drug therapy.

Furthermore, neuroinflammation is one of the important pathogenic mechanisms of PD. Our results indicate that the PD monkey model does indeed exhibit proliferation of glial cells, including astrocytes and microglia, and treatment with NES‐DA/NES can significantly reduce glial cell proliferation (Figures 6 and S2), which is crucial for the regulation of neuroinflammation in PD. When exploring the therapeutic mechanism of NES combined with MSCs, we discovered the key role of SERPINA3 in the treatment of PD. SERPINA3, as an immune‐related protein, has been identified as a biomarker in a variety of diseases, such as AD and MS [40, 41, 42]. In the CNS, the main source of SERPINA3 is astrocytes, and its expression is upregulated by IL‐1β, TNF‐a, IL‐6, and IL‐6 receptor complexes, while inhibition of SERPINA3 can improve IL‐1β‐induced inflammation [43, 44, 45, 46]. The activation of immune inflammation in the brain is one of the important causes of PD, and MPTP treatment significantly increased the expression of proinflammatory factors such as IL‐6, IL‐1β, and TNF‐a. Therefore, inflammatory factors may promote the high expression of SERPINA3 and contribute to the pathology of PD, and NES‐DN treatment significantly restored SERPINA3 levels, thereby reducing the levels of IL6, TNF‐a, and p‐p65/p65. Our results suggest that SERPINA3 may be a potential important target for NES in PD treatment.

Previous studies have shown that the problem of cell survival in vivo in stem cell transplantation has hindered the application of this therapy. Whether in autologous, allogeneic, or xenogeneic models, more than 90% of embryonic and human pluripotent stem cell‐derived dopamine neurons cannot survive after transplantation [59, 60, 61]. Large‐scale transplantation to compensate for limited survival may cause inflammatory responses due to large‐scale cell death [62, 63, 64]. The latest evidence found that p53‐mediated apoptosis is the main cause of dopamine neuron death, and TNF‐α/NF‐κB induces the upstream signaling pathway of this process. The use of TNF‐α inhibitor adalimumab can improve the survival rate of transplanted dopamine neurons in PD mouse models, and form extensive neural reinnervation and functional recovery [65]. Interestingly, in our study, NES had a significant inhibitory effect on TNF‐α/NF‐κB, and NES combined with MSCs‐induced DA neuron treatment significantly improved the improvement of PD pathology. This suggests that NES may have an important improvement in the survival or therapeutic effect of transplanted cells, which provides new guidance for the advancement of cell transplantation therapy.

This study has some limitations. First, we only explored the role of NES or NES combined with MSCs in PD, and lacked the promoting effect of NES on MSCs in PD treatment, which also deserves further exploration. Second, studies on acupuncture have shown that stimulating different acupoints in the human body can produce different effects [13]. We selected two stimulation sites for the study, which means that optimizing the selection of specific stimulation sites may yield better therapeutic effects, and this also requires further investigation. Furthermore, while we demonstrated that SERPINA3 may be a potential target for treating PD, the safety and efficacy of directly modulating SERPINA3 in a PD model require more precise evaluation. Nevertheless, our results provide valuable insights into NES and NES‐DN therapy for PD.

All in all, the efficacy of NES on PD has been proven in practice and deserves further promotion, while its therapeutic mechanism requires more in‐depth exploration. Furthermore, synergistic treatment strategies of NES combined with other therapies need to be tried, which will provide more effective effects for PD patients.

## 4 Methods

### 4.1 Animals and Ethics Statement

Adult healthy (8‐year old) male cynomolgus monkeys were housed in cages and examined for their behaviors at Guangdong Landau Biotechnology Co. Ltd., which is an Association for Assessment and Accreditation of Laboratory Animal Care‐accredited facility. All animal‐related protocols were approved in advance by the Institutional Animal Care and Use Committee (IACUC) of Guangdong Landau Biotechnology Co. Ltd and Jinan University (Ethics number: LDACU20210518‐01). This study occurred in strict compliance with the “Guide for the Care and Use of Laboratory Animals (2011)” to ensure the safety of personnel and animal welfare. Health and behavior of the monkeys were monitored daily by the husbandry staff and veterinarians. Animal information is presented in Supporting Information.

### 4.2 Isolation and Expansion of hADSCs

The adipose tissue was liposuctioned from healthy patients. The Ethics Committee of Shanghai East Hospital Affiliated to Tongji University approved the study. According to the previous research, hADSCs were primarily isolated and cultured [24]. In brief, adipose tissue was firstly rinsed by PBS 1% penicillin–streptomycin antibiotic (Gibco, Carlsbad, CA, USA), centrifuged and aspirated. The adipose tissue was digested with 0.2% collagenase A (Roche, Manheim, Germany) at 37°C for 1 h, followed by centrifugation at 1500 rpm for 10 min. The cell pellet was resuspended in Dulbecco's modified Eagle's medium (DMEM) (HyClone, GE Healthcare, Little Chalfont, UK) supplemented with 10% fetal bovine serum (FBS) (Invitrogen, California, USA) and 1% penicillin–streptomycin antibiotic (Gibco) and further filtered by 70 um filters (Pall Life Sciences, MI). After another centrifugation at 210 *g* for 10 min, the cell pellet was resuspended in the above medium and later plated onto the 10‐cm dishes (Corning, Lowell, MA), when the cells reached 80–90% confluence, they were sub‐cultured using trypsin (Gibco). hADSCs at passages 3 and 5 were used for all the following studies described.

### 4.3 hADSC Differentiated Into Mesodermal Trilineage Cells and Dopamine Neurons

The hADSCs at P3 were seeded into six‐well plates at a density of 5 × 10^3^/cm^2^ and incubated for 14–21 days in the adipogenic/osteogenic/chondrogenic culture media [26]. hADSCs were washed in PBS for three times, fixed with 4% paraformaldehyde (PFA) for 10 min, and stained respectively with Oil Red O (Sigma, USA), Alizarin bordeaux (Sigma), and Alcian blue (HUXMA‐90041; Cyagen Biosciences Inc.) at room temperature, and observed under a microscope. Dopaminergic neuronal differentiation protocol followed the published [25, 66] and was optimized according to hADSC characteristics different from iPSCs and ESCs. The differentiation ratio was calculated with five independent microscopic fields.

### 4.4 CD Surface Marker Characteristic Analysis

Fluorescently labeled antibodies anti‐CD44 (11‐0441‐81; eBioscience, USA), anti‐CD90 (14‐0909‐82; eBioscience), anti‐CD73 (12‐0731‐82; eBioscience), anti‐CD105 (17‐1051‐82; eBioscience), and anti‐CD34 (14‐0341‐82; eBioscience) were used for hADSC analysis by fluorescence‐activated cell sorting [67]. Briefly, approximately 5 × 10^4^ cells were incubated with fluorescence‐conjugated antibodies for 30 min at room temperature. Quantitative analysis was performed using C6 flow cytometry (Beckman Coulter, CA, USA), and statistical analysis was performed by FlowJo software (Tree Star Inc., Ashland, OR, USA). The representative figures were randomly chosen among three independent experimental repeats.

### 4.5 Cytoimmunostaining

The hADSC were tracked and identified by immunohistochemical staining with the neuron markers tyrosine hydroxylase antibody (TH; Novus Biologicals; NB300‐109) and DAPI Solution (Novus Biologicals; NBP2‐31156) by following previously published methods [68]. The immunocytochemistry staining was performed as follows: the hADSCs were fixed with 4% PFA in PBS for 15–20 min at RT, the cells were permeabilized with 0.25% Triton X‐100 in PBS for 10 min at RT; blocked in block solution with 3% BSA or donkey serum for 30 min, and incubated with the primary antibodies (diluted block solution) for 1 h at RT or overnight at 4°C; at the same time, the IgG isotype controls corresponding to each primary antibody were set to exclude the false‐positive detection; incubated in fluorochrome‐conjugated secondary antibodies for 1 h at RT and DAPI solution for another 10 min to stain the nuclear; ultimately mounted on a slide using antifade mounting solution. Fluorescence signals can be detected using the confocal microscope (Leica SP5) under the proper exciting wavelength. hADSC were labeled with EGFP expressing lentivirus [68] before induction into dopamine neuron like cells for 24 h and later collected to be transplantated into the brain.

### 4.6 PD Monkey Model

To comprehensively evaluate the efficacy of the NES and NES combined with MSCs therapeutic strategy, we established a unilateral parkinsonism monkey model. We first anesthetized the animals by intraperitoneal injection of atropine (0.3–0.5 mg/kg), ketamine (10–12 mg/kg) and sodium pentobarbital (15–20 mg/kg), and then fixed them on the operating table using a large animal brain stereotaxic injector (RWD68915). The MPTP (M0896; Sigma–Aldrich) solution (80 µg/µL) was directly injected into the unilateral putamen (1.6 mg) and SNpc (0.8 mg), under the guidance of MRI of the monkey [69]. For all monkeys used in the experiments, behavioral observations and PD scores were conducted on the monkeys before modeling and 1 month and 3 months after modeling.

### 4.7 Cell Transplantation

Prior to transplantation, iron oxide nanoparticles were added to hADSC‐induced DA neurons to label the cells. hADSC‐induced DA neuron suspension was prepared at 1 × 10^5^ cells per µL. For all monkey models, the cell suspension was injected under MRI guidance into two bundles (one in the putamen and one in the SNpc) along the side of the MPTP, with 50 µL injected in each bundle (total 5 × 10^6^ cells in the putamen and 2 × 10^6^ cells in the SNpc), or 50 µL 1×PBS served as control group. The monkeys were given antibiotics for 3 days after surgery and anti‐Cyclosporine A for 3 weeks with a dose of 30 mg/kg gradually decreasing to 15 mg/kg until euthanasia.

### 4.8 NES Procedures

Following our previously established standard procedures, animals were introduced to electrical stimulation after extensive chair training. To familiarize the animal with the procedures, needles were administered for two to three trials, each lasting approximately 30 min. The stimulation sites selected in this study were based on the acupoints previously studied (located by the anatomical functional areas of the body surface), including: (1) On the midline of the head, at the midpoint of the line connecting the apexes of both ears, 5 cm directly above the midpoint of the anterior hairline; and (2) On the lateral scalp, specifically in the region corresponding to the anterior and posterior boundaries of the precentral gyrus (motor cortex) projected onto the scalp surface, which exists symmetrically on both sides of the head. The electrical stimulation areas were shaven and cleaned with alcohol before the needle was inserted. The stainless‐steel needles used in the study were 0.18 mm in diameter and 20 mm in length. Electrical stimulations were supplied by using Acupuncture Stimulator model 808II (AA Health Device, Tempe, AZ). Bi‐directional square‐wave electrical pulses (0.2 ms duration, 100 Hz) were administered for approximately 30 min per NES treatment. The amplitude level was 3 mA at the beginning and then increased gradually to 4–5 mA. The animal was closely monitored by multiple video cameras. Discomfort was expressed by raised eyebrows or by withdrawal reflex or verbal expression. All animals received five NES treatment sessions per week for 3 weeks.

### 4.9 Behavioral Analysis of Motor Function

*Monkey PD score*: For all monkey behavioral tests, the behavioral paradigms were performed and observed by three trained technicians independently, who were blinded to the treatments of the monkeys. Trained personnel recorded all behaviors of the monkeys in their home cages. Refer to the Kurlan Scale to evaluate and score the following items [70, 71]: gait (0–5), tremor (0–5), body posture (0–5), global motor function (0–5), bradykinesia (0–5), balance and coordination (0–5), and defensive reaction (0–5). A score of zero represents a normal monkey, and a maximum score of 35 represents an animal with severe PD symptoms.

*Monkey behavior collection*: In order to evaluate the behavioral changes of the PD monkey model before and after treatment, we used the process of the monkey grabbing food to judge its behavioral function. The process of obtaining food was recorded with a video camera.

### 4.10 MRI Data Acquisition

MR anatomical images were acquired on a 3.0 T scanner (GE Discovery 750, Milwaukee, USA) equipped with an 8‐channel customized head coil for macaques (Medcoil MK80, Suzhou, China) at the PET/CT‐MRI center in the First Affiliated Hospital of Jinan University. Prior to each MRI scan, the monkeys underwent a fasting period of at least 6 h and were anesthetized with an intramuscular injection of ketamine (0.1–0.2 mL/kg) and atropine (0.1 mL/kg). The whole‐brain images were acquired with a 3D Bravo T1 sequence (TR = 8.4 ms, TE = 3.5 ms, slice thickness = 0.5 mm, matrix size = 300 × 300, FOV = 15 × 15 cm).

### 4.11 PET Data Acquisition and Data Analysis

Animals underwent PET with ^11^C‐CFT to assess in vivo dopamine transporter (DAT) imaging in the striatum [72, 73]. ^11^C‐CFT was produced at Center of Cyclotron and PET Radiopharmaceuticals, Department of Nuclear Medicine and PET/CT‐MRI Center, the First Affiliated Hospital of Jinan University, according to the procedures from reported literature [74]. Each monkey was first anesthetized with ketamine (0.1–0.2 mL/kg) and atropine (0.1 mL/kg) and then intravenously injected with a dose of 0.3–0.6 mCi/kg of ^11^C‐CFT. After 60 min, the monkey was placed in a homemade PET imaging head holder and underwent a 10‐min static brain PET/CT scan while in the supine position.

All data were acquired using a PET/CT system (GE Discovery Elite 690, USA). CT and PET data were collected with a slice thickness of 3.27 mm, slice interval of 3.75 mm, matrix size of 256 × 256 and scan FOV of 70 cm in 3D time‐of‐flight mode. CT data, acquired for attenuation‐corrections, were reconstructed in standard mode with DFOV of 30 cm and window width/window level 100/45, advanced statistical iterative reconstruction 40%. The PET data were attenuation‐corrected by integrated CTAC technology. PET‐MR images were coregistered by PMOD 4.1 (PMOD Technology, Switzerland) for analyzing the imaging data in the volumes of interest (VOIs), including the caudate, the putamen and the cerebellum cortical grey matter. Standard uptake values (SUVs) were calculated as SUV = [(VOI activity) × (bodyweight)]/injected dose), and the SUV ratio was calculated using the cerebellum cortical grey matter as a pseudo reference region.

### 4.12 CSF and Plasma Sample Collection

The CSF sample collection and assay followed standard protocols and have been described previously [75]. In short, after monkeys fasted for at least 8 h, a lumbar puncture was performed at the intervertebral space L3–L4 or L4–L5 using a standard needle. CSF was collected into a 1.5 mL sterile microtube (Axygen; MCT‐150‐C) and immediately frozen at −80°C. Blood samples were obtained on the same day as the lumbar puncture in fasting conditions. Whole blood was drawn with a 20 G needle gauge into a 5 mL EDTA tube. Tubes were gently inverted 5–10 times and centrifuged at 3000×*g* for 10 min at 4°C. The supernatant was aliquoted in volumes of 200 µL into EP tubes and immediately frozen at −80°C. The samples were processed at room temperature. The time between collection and freezing of both CSF and plasma sample was <30 min.

### 4.13 Immunohistochemical Analysis

Nine months after transplantation, animals were sacrificed by overdose of pentobarbital and perfused transcardially with 4% PFA in 0.1 M PBS. Brain tissues were cut into 30‐µm‐thick frozen coronal sections at the level of the posterior striatum using a cryostat. Blocking solution for fixed cells and brain sections included 10% normal donkey serum (Jackson Immunoresearch) in PBS surplus with 0.4% Triton X‐100. Primary antibodies used for immunohistochemistry were as follows: NeuN (abcam; ab177487), TH (abcam; ab76442), MAP2 (abcam; ab183830), GFP (Sigma; MAB3580), Iba1 (abcam; ab178846), and GFAP (Invitrogen; 13‐0300). Secondary antibodies were anti‐rabbit/mouse IgG conjugated to Alexa Fluor 488 or 594 (Invitrogen) and anti‐chk IgG conjugated to Alexa Fluor 647 (Invitrogen). Costaining with DAPI was also performed to confirm nuclear staining.

### 4.14 Western Blot Analysis

Tissues were ground using a grinder (Luca Sequencing Instrument Co., Ltd, Guangzhou, China) and placed in prepared protein lysis buffer (RIPA lysis buffer, protease inhibitor, and phosphatase inhibitor in a ratio of 98:1:1). The lysates were incubated on ice for 30 min, sonicated, and centrifuged at the maximum speed for 10 min. Tissues were homogenized and total protein was extracted and quantified using the BCA protein quantification kit (Solarbio, Beijing, China). Proteins were separated by electrophoresis on 4–12% sodium dodecyl sulfate‐polyacrylamide gels and transferred to polyvinylidene fluoride (PVDF) membranes. PVDF membranes were blocked with 5% nonfat dry milk for 1 h, bound with primary antibody and incubated overnight at 4°C. Primary antibodies were used: TH (Invitrogen; PA5‐85167), Vinculin (Sigma; MAB3574), GAPDH (Proteintech; 60004‐1‐Ig), MNF1 (abcam; ab126575), MNF2 (CST; 9482S), OPA1 (CST; 80471S), DRP1 (abcam; ab56788), Fis1 (CST; 32525S), NeuN (abcam; ab177487), PSD95 (CST; 2507S), synapsin‐1 (CST; 5297S), synaptophysin (CST; 25056S), Iba1 (abcam; ab178846), GFAP (Invitrogen; 13‐0300), SERPINA3 (Proteintech; 12192‐1‐AP), NF‐kB p65 (CST; 8242S), IL6 (abcam; ab233551), and TNF‐a (CST; 3707S). Finally, secondary antibodies labeled with horseradish peroxidase were incubated together. Protein bands were detected using an electrochemiluminescence kit (CLINX, 000403001A) and the results were analyzed using ImageJ software.

### 4.15 Transmission Electron Microscopy

The monkeys were anesthetized, and some putamen tissue was removed and stored in 2.5% glutaraldehyde overnight. The next day, the brain was cut into 50‐mm‐thick sections using a vibrating knife. The sections were treated in 1% cesium tetroxide solution for 1 h, dehydrated in graded ethanol, and embedded in epoxy resin. The polymerization reaction was carried out at 80°C for 24 h. The sections (60–70 nm) were stained with uranyl acetate and lead citrate and observed under a Hitachi 7100 electron microscope (Nikon, Tokyo, Japan). To quantify mitochondrial morphology, we referred to published studies and quantified the length, diameter, and area of mitochondria [76].

### 4.16 RNA‐Sequencing Analysis

Total RNA was isolated from tissues using the Trizol reagent. RNA‐seq analysis was performed at SequMed Bio Technology (Guangzhou, China) using the Illumina HiSeq X platform (Illumina, San Diego, CA, USA). We used STAR to align raw RNA‐sequencing data and further quantify the data for further analysis [77].

### 4.17 ELISA Test for DA Level

Dopamine concentration of CSF and brain tissue homogenate assayed by dopamine ELISA Kit (JM‐10911M1).

### 4.18 HMC3 Cell Culture

HMC3 cells were obtained from the American Type Culture Collection and grown in DMEM medium (Gibco). All cultures were supplemented with 10 % FBS (Gemini) and 1 % penicillin streptomycin (Gibco) at 37°C, 5% CO_2_ in a humidified incubator.

SERPINA3 plasmids were produced by Guangzhou IGE Biotechnology Co., Ltd. For plasmids transfection, cells were grown overnight to reach 70 % confluence before transfection using Lipofectamine 3000 (L3000075; Thermo Scientific) according the manufacturer's instructions, followed by the indicated treatment. 100 nm LPS (Sigma; CAS#93572‐42‐0) or 100 µm MPP^+^ (MCE; CAS#36913‐39‐0) was used to treat HMC3 cells as a positive control group.

### 4.19 Statistical Procedures

Statistical analysis was performed with GraphPad Prism 9.3.1 (GraphPad Software Inc.). Values were expressed as mean ± SD. Data were analyzed using ANOVA. Differences among means were further analyzed by posthoc multiple comparisons. The threshold for statistical significance was *p* < 0.05.

## Author Contributions

S.Y. and J.Z. conceived the research, designed the experiments, interpreted the data, and revised the manuscript. C.H.H. performed the experiments, generated the figures, analyzed the RNA sequencing experiments, and wrote the manuscript. S.E.G. provided hADCs, performed the experiments, and generated the figures. X.Z., J.X.W., X.C.S., Y.Q.L., J.H.F., Y.Q.L., and W.W. performed the experiments. C.J.L., J.H.W., J.L.G., J.Z.S., and C.X.S. harvested the tissues. J.W.L. and Y.Z.C. analyzed the RNA sequencing experiments. H.Y.W., L.W., K.L., and H.X. collected and analyzed the MRI and PET data.

All authors have read and approved the final manuscript.

## Funding

This work was supported by the National Key Research and Development Program of China (2021YFA0805300 to S.Y.), the National Natural Science Foundation of China (82171244 to S.Y., 32470564 to S.Y., 81873363 to J.Z.), Guangzhou Key Laboratory for Germ‐Free Animals and Microbiota Application (202201020381 to J.Z.), the Traditional Chinese Medicine Bureau of Guangdong Province (20242016 to J.Z.), the Ministry of Science and Technology of China (2017YFE0121100 to S.G.); Yunnan Characteristic Plant Extraction Laboratory (YKKF2023009 to S.G.), Guangzhou Basic Research Program (SL2024A03J00780 to S.Y.), Science and Technology Project in Guangzhou (202102070001 to S.Y.), the Postdoctoral Fellowship Program of CPSF (GZC20231064 to X.Z.), China Postdoctoral Science Foundation (2024M761345 to X.Z.), Scientific Research Project of Southern Medical University Stomatological Hospital (PY2023004 to X.Z.), and Medical Scientific Research Foundation of Guangdong Province (B2025678 to X.Z.).

## Ethics Statement

All animal‐related protocols were approved in advance by the Institutional Animal Care and Use Committee (IACUC) of Guangdong Landau Biotechnology Co. Ltd and Jinan University (Ethics number: LDACU20210518‐01). This study occurred in strict compliance with the “Guide for the Care and Use of Laboratory Animals (2011)” to ensure the safety of personnel and animal welfare.

## Conflicts of Interest

Author Yaqun Lu is an employee in QuanYan Biotechnology Limited Company, but has no potential relevant financial or nonfinancial interests to disclose. Author Jun Zhang is an employee in Shenzhen LUZE Biological Technology Co., Ltd., but has no potential relevant financial or nonfinancial interests to disclose. The other authors have no conflicts of interest to declare.

## Supporting information



Movie S1. Unilateral injection of MPTP leads to loss of left hand flexibility.

Movie S2. NES treatment improved left hand flexibility loss caused by MPTP injection.

Movie S3. NES‐DN treatment improved left hand flexibility loss caused by MPTP injection.

**Figure S1** Characterization of hADSCs and their differentiation into dopaminergic neuron like cells. Panel A shows hADSCs positively express mesenchymal stem cell CD markers of CD44, CD73, CD 90, and CD105 (>97%). Panel B shows hADSCs could be conditionally induced into trileage mesodermal cells of adipocyte, osteoblast and chondrocyte. Panel C shows hADSCs could be gradually induced into dopaminergic neuron like (TH positive) cells. Panel D shows the statistical analysis for their trileage differentiation ratio, CD marker positive ratio and TH positive ratio at day 3 and 7 respectively. Scale bar = 100 µm, *n* = 5. (E) Characterization of EGFP labeled hADSCs by lentivirus and their differentiation into dopaminergic neuron like cells at 24 h before transplantation. Scale bar = 100 µm.
**Figure S2** NES and NES‐DN treatments regulated the level of neuroinflammation in substantia nigra. (A) Representative images of Iba1 and GFAP immunofluorescence staining in substantia nigra, scale bar = 20 µm. (B and C) Quantitative statistics of Iba1 (B) and GFAP (C) positive cells. WT (*n* = 3), MPTP (*n* = 3), NES (*n* = 2), and NES‐DN (*n* = 3), ***p < 0.005, ****p < 0.001.
**Figure S3** Changes in SERPINA3 after NES and NES‐DN treatment. (A) The common intersection gene heatmap of MPTP_VS‐WT, NES‐VD_MPTP, and NES‐DN_VS‐MPTP. (B) Statistical chart of RNA level changes in SERPINA3. (C) QPCR results display SERPINA3 RNA quantification statistical chart. WT (*n* = 3), MPTP (*n* = 3), NES (*n* = 2), and NES‐DN (*n* = 3), **p < 0.01, ***p < 0.005. (D–F) Volcano map of MPTP_ VS_ WT (D), NES‐DN_ VS_ WT (E), and NES_ VS_ WT (F).
**Figure S4** MPP^+^ treatment or overexpression of SERPINA3 can induce an inflammatory phenotype in HMC3. (A) Schematic diagram of MPP^+^ processing or overexpression of SERPINA3 in HMC3. (B–D) Expression levels of SERPINA3 (B), IL‐1β (C), and IL‐6 (D) after MPP^+^ and LPS treatment. Ctrl (*n* = 5), MPP^+^ (*n* = 5), and LPS (*n* = 5), **p < 0.01, ***p < 0.005, ****p < 0.001. (E–G) Overexpression of SERPINA3 leads to increased expression levels of SERPINA3 (E), IL‐1β (F), and IL‐6 (G). Ctrl (*n* = 3), GFP (*n* = 3), LPS (*n* = 3), and SERPINA3 (*n* = 3), *p < 0.05, **p < 0.01, ***p < 0.005, ****p < 0.001.

## Data Availability

The raw data used and/or analyzed during the current study are available from the corresponding author on reasonable request. The RNA‐seq data are publicly available in the NGDC‐NGDC database under accession number PRJCA050274.

## References

[1] W. Poewe , K. Seppi , C. M. Tanner , et al., “Parkinson Disease,” Nature Reviews Disease Primers 3 (2017): 17013. 10.1038/nrdp.2017.13 28332488

[2] Y. Ben‐Shlomo , S. Darweesh , J. Llibre‐Guerra , et al., “The Epidemiology of Parkinson's Disease,” The Lancet 403, no. 10423 (2024): 283–292. 10.1016/S0140-6736(23)01419-8 PMC11123577 38245248

[3] H. Ye , L. A. Robak , M. Yu , M. Cykowski , and J. M. Shulman , “Genetics and Pathogenesis of Parkinson's Syndrome,” Annual Review of Pathology: Mechanisms of Disease 18, no. 1 (2023): 95–121. 10.1146/annurev-pathmechdis-031521-034145 PMC10290758 36100231

[4] M. J. Armstrong and M. S. Okun , “Diagnosis and Treatment of Parkinson Disease: A Review,” Jama 323, no. 6 (2020): 548–560. 32044947 10.1001/jama.2019.22360

[5] A. M. Lozano , J. Dostrovsky , R. Chen , and P. Ashby , “Deep Brain Stimulation for Parkinson's disease: Disrupting the Disruption,” The Lancet Neurology 1, no. 4 (2002): 225–231. 12849455 10.1016/s1474-4422(02)00101-1

[6] A. E. Lang and H. Widner , “Deep Brain Stimulation for Parkinson's disease: Patient Selection and Evaluation,” Movement Disorders 17, no. S3 (2002): S94–S101. 11948762 10.1002/mds.10149

[7] A. D. Wu , F. Fregni , D. K. Simon , C. Deblieck , and A. Pascual‐Leone , “Noninvasive Brain Stimulation for Parkinson's Disease and Dystonia,” Neurotherapeutics 5, no. 2 (2008): 345–361. 18394576 10.1016/j.nurt.2008.02.002 PMC3270324

[8] W. Zhang , B. Deng , F. Xie , et al., “Efficacy of Repetitive Transcranial Magnetic Stimulation in Parkinson's disease: A Systematic Review and Meta‐analysis of Randomised Controlled Trials,” Eclinicalmedicine 52 (2022): 101589. 35923424 10.1016/j.eclinm.2022.101589 PMC9340539

[9] L. Dinkelbach , M. Brambilla , R. Manenti , and A.‐K. Brem , “Non‐invasive Brain Stimulation in Parkinson's Disease: Exploiting Crossroads of Cognition and Mood,” Neuroscience & Biobehavioral Reviews 75 (2017): 407–418. 28119070 10.1016/j.neubiorev.2017.01.021

[10] A. A. Rabinstein and L. M. Shulman , “Acupuncture in Clinical Neurology,” The Neurologist 9, no. 3 (2003): 137–148. 12808410 10.1097/00127893-200305000-00002

[11] L. M. Shulman , X. Wen , W. J. Weiner , et al., “Acupuncture Therapy for the Symptoms of Parkinson's Disease,” Movement Disorders 17, no. 4 (2002): 799–802. 12210879 10.1002/mds.10134

[12] B.‐Y. Zeng , S. Salvage , and P. Jenner , “Current Development of Acupuncture Research in Parkinson's Disease,” Neurobiology of Acupuncture 111 (2013): 141–158. 10.1016/B978-0-12-411545-3.00007-9 24215921

[13] J.‐Q. Fan , W.‐J. Lu , W.‐Q. Tan , W.‐C. Feng , and L.‐X. Zhuang , “Acupuncture for Parkinson's Disease: From Theory to Practice,” Biomedicine and Pharmacotherapy 149 (2022): 112907. 35366533 10.1016/j.biopha.2022.112907

[14] B.‐Y. Zeng and K. Zhao , “Effect of Acupuncture on the Motor and Nonmotor Symptoms in Parkinson's Disease—A Review of Clinical Studies,” CNS Neuroscience & Therapeutics 22, no. 5 (2016): 333–341. 26843036 10.1111/cns.12507 PMC6492822

[15] J. Q. Yin , J. Zhu , and J. A. Ankrum , “Manufacturing of Primed Mesenchymal Stromal Cells for Therapy,” Nature Biomedical Engineering 3, no. 2 (2019): 90–104. 10.1038/s41551-018-0325-8 30944433

[16] M. Ghasemi , E. Roshandel , M. Mohammadian , et al., “Mesenchymal Stromal Cell‐derived Secretome‐based Therapy for Neurodegenerative Diseases Overview of Clinical Trials,” Stem Cell Research & Therapy 14, no. 1 (2023): 122. 37143147 10.1186/s13287-023-03264-0 PMC10161443

[17] Y.‐S. Zeng , Y. Ding , H.‐Y. Xu , et al., “Electro‐acupuncture and Its Combination With Adult Stem Cell Transplantation for Spinal Cord Injury Treatment a Summary of Current Laboratory Findings and a Review of Literature,” CNS Neuroscience & Therapeutics 28, no. 5 (2022): 635–647. 35174644 10.1111/cns.13813 PMC8981476

[18] T. E. Salazar , M. R. Richardson , and E. Beli , “Electroacupuncture Promotes CNS‐dependent Release of Mesenchymal Stem Cells,” Stem Cells 35, no. 5 (2017): 1303–1315. 28299842 10.1002/stem.2613 PMC5530374

[19] C. Yang , Y. Qiu , Y. Qing , et al., “Synergistic Effect of Electric Stimulation and Mesenchymal Stem Cells Against Parkinson's disease,” Aging 12, no. 16 (2020): 16062–16071. 32836217 10.18632/aging.103477 PMC7485716

[20] M. F. Beal , “Experimental Models of Parkinson's Disease,” Nature Reviews Neuroscience 2, no. 5 (2001): 325–332. 11331916 10.1038/35072550

[21] S. Schildknecht , D. A. Di Monte , R. Pape , K. Tieu , and M. Leist , “Tipping Points and Endogenous Determinants of Nigrostriatal Degeneration by MPTP,” Trends in Pharmacological Sciences 38, no. 6 (2017): 541–555. 28442167 10.1016/j.tips.2017.03.010

[22] J. P. Capitanio and M. E. Emborg , “Contributions of Non‐human Primates to Neuroscience Research,” Lancet 371, no. 9618 (2008): 1126–1135. 18374844 10.1016/S0140-6736(08)60489-4

[23] E. Bezard , C. Imbert , X. Deloire , B. Bioulac , and C. E. Gross , “A Chronic MPTP Model Reproducing the Slow Evolution of Parkinson's Disease Evolution of Motor Symptoms in the Monkey,” Brain Research 766, no. 1‐2 (1997): 107–112. 9359593 10.1016/s0006-8993(97)00531-3

[24] F. Yu , N. Witman , D. Yan , et al., “Human Adipose‐derived Stem Cells Enriched With VEGF‐modified mRNA Promote Angiogenesis and Long‐term Graft Survival in a Fat Graft Transplantation Model,” Stem Cell Research & Therapy 11, no. 1 (2020): 490. 33213517 10.1186/s13287-020-02008-8 PMC7678328

[25] M. Wei , S. Li , Z. Yang , et al., “Tetrahedral DNA Nanostructures Functionalized by Multivalent microRNA132 Antisense Oligonucleotides Promote the Differentiation of Mouse Embryonic Stem Cells Into Dopaminergic Neurons,” Nanomedicine 34 (2021): 102375. 33617970 10.1016/j.nano.2021.102375

[26] Y. Jin , J. Wang , H. Li , et al., “Extracellular Vesicles Secreted by Human Adipose‐derived Stem Cells (hASCs) Improve Survival Rate of Rats With Acute Liver Failure by Releasing lncRNA H19,” EBioMedicine 34 (2018): 231–242. 30077720 10.1016/j.ebiom.2018.07.015 PMC6116414

[27] J.‐Q. Fan , W.‐J. Lu , and W.‐Q. Tan , “Effectiveness of Acupuncture for Anxiety among Patients with Parkinson Disease: A Randomized Clinical Trial,” JAMA Network Open 5, no. 9 (2022): e2232133. 36129711 10.1001/jamanetworkopen.2022.32133 PMC9494193

[28] H. Noh , S. Kwon , S.‐Y. Cho , et al., “Effectiveness and Safety of Acupuncture in the Treatment of Parkinson's Disease a Systematic Review and Meta‐analysis of Randomized Controlled Trials,” Complementary Therapies in Medicine 34 (2017): 86–103. 28917379 10.1016/j.ctim.2017.08.005

[29] D. Weintraub , D. Aarsland , K. R. Chaudhuri , et al., “The Neuropsychiatry of Parkinson's Disease: Advances and Challenges,” Lancet Neurology 21, no. 1 (2022): 89–102. 34942142 10.1016/S1474-4422(21)00330-6 PMC8800169

[30] K. Li , S. Xu , R. Wang , et al., “Electroacupuncture for Motor Dysfunction and Constipation in Patients With Parkinson's Disease a Randomised Controlled Multi‐centre Trial,” Eclinicalmedicine 56 (2023): 101814. 36691434 10.1016/j.eclinm.2022.101814 PMC9860357

[31] S. P. Markey and N. R. Schmuff , “The Pharmacology of the Parkinsonian Syndrome Producing Neurotoxin MPTP (1–methyl‐4‐phenyl‐1,2,3,6‐tetrahydropyridine) and Structurally Related Compounds,” Medicinal Research Reviews 6, no. 4 (1986): 389–429. 3534484 10.1002/med.2610060402

[32] Q. Hu and G. Wang , “Mitochondrial Dysfunction in Parkinson's Disease,” Translational Neurodegeneration 5 (2016): 14. 27453777 10.1186/s40035-016-0060-6 PMC4957882

[33] J. W. Langston , P. Ballard , J. W. Tetrud , and I. Irwin , “Chronic Parkinsonism in Humans due to a Product of Meperidine‐analog Synthesis,” Science 219, no. 4587 (1983): 979–980. 6823561 10.1126/science.6823561

[34] J.‐S. Han , “Acupuncture: Neuropeptide Release Produced by Electrical Stimulation of Different Frequencies,” Neuron 26, no. 1 (2003): 17–22. 10.1016/s0166-2236(02)00006-1 12495858

[35] H.‐C. Lai , Q.‐Y. Chang , and C.‐L. Hsieh , “Signal Transduction Pathways of Acupuncture for Treating some Nervous System Diseases,” Evidence‐Based Complementary and Alternative Medicine 2019 (2019): 1–37. 10.1155/2019/2909632 PMC6657648 31379957

[36] M. G. Tansey , R. L. Wallings , M. C. Houser , et al., “Inflammation and Immune Dysfunction in Parkinson disease,” Nature Reviews Immunology 22, no. 11 (2022): 657–673. 10.1038/s41577-022-00684-6 PMC8895080 35246670

[37] J.‐H. Jang , M.‐J. Yeom , S. Ahn , et al., “Acupuncture Inhibits Neuroinflammation and Gut Microbial Dysbiosis in a Mouse Model of Parkinson's Disease,” Brain, Behavior, and Immunity 89 (2020): 641–655. 32827699 10.1016/j.bbi.2020.08.015

[38] X. Ma , Q. Wang , W. Yuan , et al., “Electroacupuncture Alleviates Neuroinflammation and Motor Dysfunction by Regulating Intestinal Barrier Function in a Mouse Model of Parkinson Disease,” Journal of Neuropathology & Experimental Neurology 80, no. 9 (2021): 844–855. 34343334 10.1093/jnen/nlab046

[39] Y.‐Y. Xin , J.‐X. Wang , and A.‐J. Xu , “Electroacupuncture Ameliorates Neuroinflammation in Animal Models,” Acupuncture in Medicine 40, no. 5 (2022): 474–483. 35229660 10.1177/09645284221076515

[40] K. A. Walker , J. Chen , L. Shi , et al., “Proteomics Analysis of Plasma From Middle‐aged Adults Identifies Protein Markers of Dementia Risk in Later Life,” Science Translational Medicine 15, no. 705 (2023): eadf5681. 37467317 10.1126/scitranslmed.adf5681 PMC10665113

[41] Z. Fan , Y. Gao , N. Jiang , et al., “Immune‐related SERPINA3 as a Biomarker Involved in Diabetic Nephropathy Renal Tubular Injury,” Frontiers in Immunology 13 (2022): 979995. 36304455 10.3389/fimmu.2022.979995 PMC9592916

[42] N. Fissolo , C. Matute‐Blanch , M. Osman , et al., “CSF SERPINA3 Levels Are Elevated in Patients with Progressive MS,” Neurology Neuroimmunology & Neuroinflammation 8, no. 2 (2021): e941. 33436375 10.1212/NXI.0000000000000941 PMC8105904

[43] S. Das and H. Potter , “Expression of the Alzheimer Amyloid‐promoting Factor Antichymotrypsin Is Induced in human Astrocytes by IL‐1,” Neuron 14, no. 2 (1995): 447–456. 7857652 10.1016/0896-6273(95)90300-3

[44] T. Kordula , M. Bugno , R. E. Rydel , and J. Travis , “Mechanism of Interleukin‐1‐ and Tumor Necrosis Factor Alpha‐dependent Regulation of the Alpha 1‐antichymotrypsin Gene in human Astrocytes,” The Journal of Neuroscience 20, no. 20 (2000): 7510–7516. 11027208 10.1523/JNEUROSCI.20-20-07510.2000 PMC6772857

[45] Z. Liu , R. Liu , R. Wang , et al., “Sinensetin Attenuates IL‐1β‐induced Cartilage Damage and Ameliorates Osteoarthritis by Regulating SERPINA3,” Food & Function 13, no. 19 (2022): 9973–9987. 36056701 10.1039/d2fo01304e

[46] C. R. Abraham , D. J. Selkoe , and H. Potter , “Immunochemical Identification of the Serine Protease Inhibitor Alpha 1‐antichymotrypsin in the Brain Amyloid Deposits of Alzheimer's Disease,” Cell 52, no. 4 (1988): 487–501. 3257719 10.1016/0092-8674(88)90462-x

[47] N. D. Schiff , J. T. Giacino , C. R. Butson , et al., “Thalamic Deep Brain Stimulation in Traumatic Brain Injury: A Phase 1, Randomized Feasibility Study,” Nature Medicine 29, no. 12 (2023): 3162–3174. 10.1038/s41591-023-02638-4 PMC11087147 38049620

[48] K. J. Clancy , J. A. Andrzejewski , Y. You , et al., “Transcranial Stimulation of Alpha Oscillations Up‐regulates the Default Mode Network,” Proceedings of the National Academy of Sciences of the United States of America 119, no. 1 (2021): e2110868119. 10.1073/pnas.2110868119 PMC8740757 34969856

[49] J. K. Krauss , N. Lipsman , T. Aziz , et al., “Technology of Deep Brain Stimulation: Current Status and Future Directions,” Nature Reviews Neurology 17, no. 2 (2020): 75–87. 33244188 10.1038/s41582-020-00426-z PMC7116699

[50] T. Milekovic , E. M. Moraud , N. Macellari , et al., “A Spinal Cord Neuroprosthesis for Locomotor Deficits due to Parkinson's Disease,” Nature Medicine 29, no. 11 (2023): 2854–2865. 10.1038/s41591-023-02584-1 37932548

[51] D. M. Hoang , P. T. Pham , T. Q. Bach , et al., “Stem Cell‐based Therapy for human Diseases,” Signal Transduction and Targeted Therapy 7, no. 1 (2022): 272. 35933430 10.1038/s41392-022-01134-4 PMC9357075

[52] Y.‐K. Wang , W.‐W. Zhu , M.‐H. Wu , et al., “Human Clinical‐Grade Parthenogenetic ESC‐Derived Dopaminergic Neurons Recover Locomotive Defects of Nonhuman Primate Models of Parkinson's Disease,” Stem Cell Reports 11, no. 1 (2018): 171–182. 29910127 10.1016/j.stemcr.2018.05.010 PMC6067059

[53] A. Morizane , T. Kikuchi , T. Hayashi , et al., “MHC Matching Improves Engraftment of iPSC‐derived Neurons in Non‐human Primates,” Nature Communications 8, no. 1 (2017): 385. 10.1038/s41467-017-00926-5 PMC5577234 28855509

[54] A. Uccelli , L. Moretta , and V. Pistoia , “Mesenchymal Stem Cells in Health and Disease,” Nature Reviews Immunology 8, no. 9 (2008): 726–736. 10.1038/nri2395 19172693

[55] H. Gao and W. Ding , “Effect and Mechanism of Acupuncture on Endogenous and Exogenous Stem Cells in Disease Treatment: A Therapeutic Review,” Life Sciences 331 (2023): 122031. 37598978 10.1016/j.lfs.2023.122031

[56] Y. Geng , D. Chen , J. Zhou , et al., “Synergistic Effects of Electroacupuncture and Mesenchymal Stem Cells on Intestinal Ischemia/Reperfusion Injury in Rats,” Inflammation 39, no. 4 (2016): 1414–1420. 27221138 10.1007/s10753-016-0373-8

[57] S. M. Ahn , Y. R. Kim , Y.‐I. Shin , et al., “Therapeutic Potential of a Combination of Electroacupuncture and TrkB‐Expressing Mesenchymal Stem Cells for Ischemic Stroke,” Molecular Neurobiology 56, no. 1 (2018): 157–173. 29682700 10.1007/s12035-018-1067-z

[58] Y. Yang , H. Y. Xu , Q. W. Deng , et al., “Electroacupuncture Facilitates the Integration of a Grafted TrkC‐modified Mesenchymal Stem Cell‐derived Neural Network Into Transected Spinal Cord in Rats via Increasing Neurotrophin‐3,” CNS Neuroscience & Therapeutics 27, no. 7 (2021): 776–791. 33763978 10.1111/cns.13638 PMC8193704

[59] T.‐Y. Park , J. Jeon , N. Lee , et al., “Co‐transplantation of Autologous Treg Cells in a Cell Therapy for Parkinson's disease,” Nature 619, no. 7970 (2023): 606–615. 37438521 10.1038/s41586-023-06300-4 PMC12012854

[60] C. E. Sortwell , M. R. Pitzer , and T. J. Collier , “Time Course of Apoptotic Cell Death Within Mesencephalic Cell Suspension Grafts: Implications for Improving Grafted Dopamine Neuron Survival,” Experimental Neurology 165, no. 2 (2000): 268–277. 10993687 10.1006/exnr.2000.7476

[61] M. Emgård , J. Karlsson , O. Hansson , and P. Brundin , “Patterns of Cell Death and Dopaminergic Neuron Survival in Intrastriatal Nigral Grafts,” Experimental Neurology 160, no. 1 (1999): 279–288. 10630212 10.1006/exnr.1999.7198

[62] C. Winkler , D. Kirik , and A. Björklund , “Cell Transplantation in Parkinson's Disease: How Can We Make It Work?,” Trends in Neurosciences 28, no. 2 (2005): 86–92. 15667931 10.1016/j.tins.2004.12.006

[63] S. Kriks , J.‐W. Shim , J. Piao , et al., “Dopamine Neurons Derived From human ES Cells Efficiently Engraft in Animal Models of Parkinson's disease,” Nature 480, no. 7378 (2011): 547–551. 22056989 10.1038/nature10648 PMC3245796

[64] Y. Tao , S. C. Vermilyea , M. Zammit , et al., “Autologous Transplant Therapy Alleviates Motor and Depressive Behaviors in parkinsonian Monkeys,” Nature Medicine 27, no. 4 (2021): 632–639. 10.1038/s41591-021-01257-1 PMC8198752 33649496

[65] T. W. Kim , S. Y. Koo , M. Riessland , et al., “TNF‐NF‐κB‐p53 Axis Restricts in Vivo Survival of hPSC‐derived Dopamine Neurons,” Cell 187, no. 14 (2024): 3671–3689.e3623. 38866017 10.1016/j.cell.2024.05.030 PMC11641762

[66] M. G. Otero , S. Bell , A. H. Laperle , et al., “Organ‐Chips Enhance the Maturation of Human iPSC‐Derived Dopamine Neurons,” International Journal of Molecular Sciences 24, no. 18 (2023): 14227. 37762529 10.3390/ijms241814227 PMC10531789

[67] F. Zhou , S. Gao , L. Wang , et al., “Human Adipose‐derived Stem Cells Partially Rescue the Stroke Syndromes by Promoting Spatial Learning and Memory in Mouse Middle Cerebral Artery Occlusion Model,” Stem Cell Research & Therapy 6, no. 1 (2015): 92. 25956259 10.1186/s13287-015-0078-1 PMC4453264

[68] S. Gao , P. Zhao , C. Lin , et al., “Differentiation of human Adipose‐derived Stem Cells Into Neuron‐Like Cells Which Are Compatible With Photocurable Three‐dimensional Scaffolds,” Tissue Engineering Part A 20, no. 7‐8 (2014): 1271–1284. 24251600 10.1089/ten.tea.2012.0773 PMC3993073

[69] T. Hayashi , S. Wakao , M. Kitada , et al., “Autologous Mesenchymal Stem Cell–derived Dopaminergic Neurons Function in parkinsonian Macaques,” Journal of Clinical Investigation 123, no. 1 (2012): 272–284. 23202734 10.1172/JCI62516 PMC3533267

[70] R. D. Smith , Z. Zhang , R. Kurlan , M. McDermott , and D. M. Gash , “Developing a Stable Bilateral Model of Parkinsonism in rhesus Monkeys,” Neuroscience 52, no. 1 (1993): 7–16. 8433810 10.1016/0306-4522(93)90176-g

[71] R. Kurlan , M. H. Kim , and D. M. Gash , “Oral Levodopa Dose‐response Study in MPTP‐induced Hemiparkinsonian Monkeys Assessment With a New Rating Scale for Monkey Parkinsonism,” Movement Disorders 6, no. 2 (1991): 111–118. 2057003 10.1002/mds.870060205

[72] Y. Takagi , J. Takahashi , H. Saiki , et al., “Dopaminergic Neurons Generated From Monkey Embryonic Stem Cells Function in a Parkinson Primate Model,” Journal of Clinical Investigation 115, no. 1 (2005): 102–109. 15630449 10.1172/JCI21137 PMC539189

[73] H. Saiki , T. Hayashi , R. Takahashi , and J. Takahashi , “Objective and Quantitative Evaluation of Motor Function in a Monkey Model of Parkinson's disease,” Journal of Neuroscience Methods 190, no. 2 (2010): 198–204. 20488205 10.1016/j.jneumeth.2010.05.009

[74] K. Någren , L. Müller , C. Halldin , C. G. Swahn , and P. Lehikoinen , “Improved Synthesis of some Commonly Used PET Radioligands by the Use of [11C]Methyl Triflate,” Nuclear Medicine and Biology 22, no. 2 (1995): 235–239. 7767319 10.1016/0969-8051(94)00083-v

[75] Z. Tu , S. Yan , B. Han , et al., “Tauopathy Promotes Spinal Cord‐dependent Production of Toxic Amyloid‐beta in Transgenic Monkeys,” Signal Transduction and Targeted Therapy 8, no. 1 (2023): 358. 37735155 10.1038/s41392-023-01601-6 PMC10514290

[76] E. F. Fang , Y. Hou , K. Palikaras , et al., “Mitophagy Inhibits Amyloid‐β and Tau Pathology and Reverses Cognitive Deficits in Models of Alzheimer's Disease,” Nature Neuroscience 22, no. 3 (2019): 401–412. 30742114 10.1038/s41593-018-0332-9 PMC6693625

[77] A. Dobin , C. A. Davis , F. Schlesinger , et al., “STAR: Ultrafast Universal RNA‐seq Aligner,” Bioinformatics 29, no. 1 (2013): 15–21. 23104886 10.1093/bioinformatics/bts635 PMC3530905

