# Supplementary material for: Therapeutic Effects of Noninvasive Electrical Stimulation in Combination Transplantation of Human Adipose‐Derived Stem Cells‐Derived Dopaminergic Neuron on the Monkey Model of Parkinson's Disease
Source: MedComm (2020). 2026 Jan 28;7(2):e70595. doi: 10.1002/mco2.70595 (PMC12848770; doi:10.1002/mco2.70595)
Supplement: Supplementary file 4 — Figure S1 Characterization of hADSCs and their differentiation into dopaminergic neuron like cells. Panel A shows hADSCs positively express mesenchymal stem cell CD markers of CD44, CD73, CD 90, and CD105 (>97%). Panel B shows hADSCs could be conditionally induced into trileage mesodermal cells of adipocyte, osteoblast and chondrocyte. Panel C shows hADSCs could be gradually induced into dopaminergic neuron like (TH positive) cells. Panel D shows the statistical analysis for their trileage differentiation ratio, CD marker positive ratio and TH positive ratio at day 3 and 7 respectively. Scale bar = 100 µm, n = 5. (E) Characterization of EGFP labeled hADSCs by lentivirus and their differentiation into dopaminergic neuron like cells at 24 h before transplantation. Scale bar = 100 µm. Figure S2 NES and NES‐DN treatments regulated the level of neuroinflammation in substantia nigra. (A) Representative images of Iba1 and GFAP immunofluorescence staining in substantia nigra, scale bar = 20 µm. (B and C) Quantitative statistics of Iba1 (B) and GFAP (C) positive cells. WT (n = 3), MPTP (n = 3), NES (n = 2), and NES‐DN (n = 3), ***p < 0.005, ****p < 0.001. Figure S3 Changes in SERPINA3 after NES and NES‐DN treatment. (A) The common intersection gene heatmap of MPTP_VS‐WT, NES‐VD_MPTP, and NES‐DN_VS‐MPTP. (B) Statistical chart of RNA level changes in SERPINA3. (C) QPCR results display SERPINA3 RNA quantification statistical chart. WT (n = 3), MPTP (n = 3), NES (n = 2), and NES‐DN (n = 3), **p < 0.01, ***p < 0.005. (D–F) Volcano map of MPTP_ VS_ WT (D), NES‐DN_ VS_ WT (E), and NES_ VS_ WT (F). Figure S4 MPP+ treatment or overexpression of SERPINA3 can induce an inflammatory phenotype in HMC3. (A) Schematic diagram of MPP+ processing or overexpression of SERPINA3 in HMC3. (B–D) Expression levels of SERPINA3 (B), IL‐1β (C), and IL‐6 (D) after MPP+ and LPS treatment. Ctrl (n = 5), MPP+ (n = 5), and LPS (n = 5), **p < 0.01, ***p < 0.005, ****p < 0.001. (E–G) Overexpression of SERPI [file MCO2-7-e70595-s003.docx]

# Ttitle page:

# Therapeutic effects of non-invasive electrical stimulation in combination transplantation of human adipose-derived stem cells-derived dopaminergic neuron on the monkey model of Parkinson’s disease

**Running title: NES treatment for MPTP monkey**

Chunhui Huang^1#^, Shane Gao^2#^, Xiao Zheng^4#^, Xichen Song^1#^, Jiaxi Wu^1^, Kai Liao^6^, Jiawei Li^1^, Yingqi Lin^1^, Caijuan Li^1^, Yaqun Lu^3,7^, Jiahao Feng^8^, Huiyi Wei^6^, Lu Wang^6^, Hao Xu^6^, Wei Wang^1^, Yizhi Chen^1^, Jianhao Wu^1^, Jiale Gao^1^, Junzhu Song^1^, Chunxiang Shi^1^, Jun Zhang^1,3,9^*, Sen Yan^1,5^*

^1^The Sixth Affiliated Hospital of Jinan University, Dongguan, 523710, China; Guangdong Provincial Key Laboratory of Non-human Primate Research, Guangdong-Hong Kong-Macau Institute of CNS Regeneration, Jinan University, Guangzhou, 510632, China; School of Traditional Chinese Medicine, Jinan University, Guangzhou, 510632, Guangdong, China

^2^Department of neurosurgery, Shanghai East Hospital, School of Medicine，Tongji University, Shanghai, 200120, China

^3^Department of Traditional Chinese Medicine, The First Affiliated Hospital of Jinan University, Guangzhou, 510630, China

^4^Stomatological Hospital, School of Stomatology, Southern Medical University, Guangzhou, 510280, China

^5^State Key Laboratory of Bioactive Molecules and Druggability Assessment, Jinan University, Guangzhou, 510632, Guangdong, China

^6^Department of Nuclear Medicine and PET/CT-MRI Center, The First Affiliated Hospital of Jinan University, Guangzhou, 510630, China

^7^QuanYan Biotechnology Limited Company, Shanghai, 200012, China

^8^School of Medicine, Sun Yat-Sen University, Guangzhou, China

^9^Heilongjiang University of Chinese Medicine, Heilongjiang, 150040, China

Chunhui Huang, Shane Gao, Xiao Zheng and Xichen Song have contributed equally to this work.

Corresponding author: Jun Zhang (e-mail: [zhangjun@jnu.edu.cn](mailto:zhangjun@jnu.edu.cn)) and Sen Yan (e-mail: [231yansen@163.com](mailto:231yansen@163.com))

**Ethics approval:** All animal-related protocols were approved in advance by the Institutional Animal Care and Use Committee (IACUC) of Guangdong Landau Biotechnology Co. Ltd and Jinan University (Ethics number: LDACU20210518-01). This study occurred in strict compliance with the “Guide for the Care and Use of Laboratory Animals (2011)” to ensure the safety of personnel and animal welfare.

**Supplemental Information**

Including:

Supplementary Figure S1-S4 and figure legends.

Supplementary Movie 1. Unilateral injection of MPTP leads to loss of left hand flexibility.

Supplementary Movie 2. NES treatment improved left hand flexibility loss caused by MPTP injection.

Supplementary Movie 3. NES-DN treatment improved left hand flexibility loss caused by MPTP injection.

**Supplementary Figure S1-S4 and figure legends**


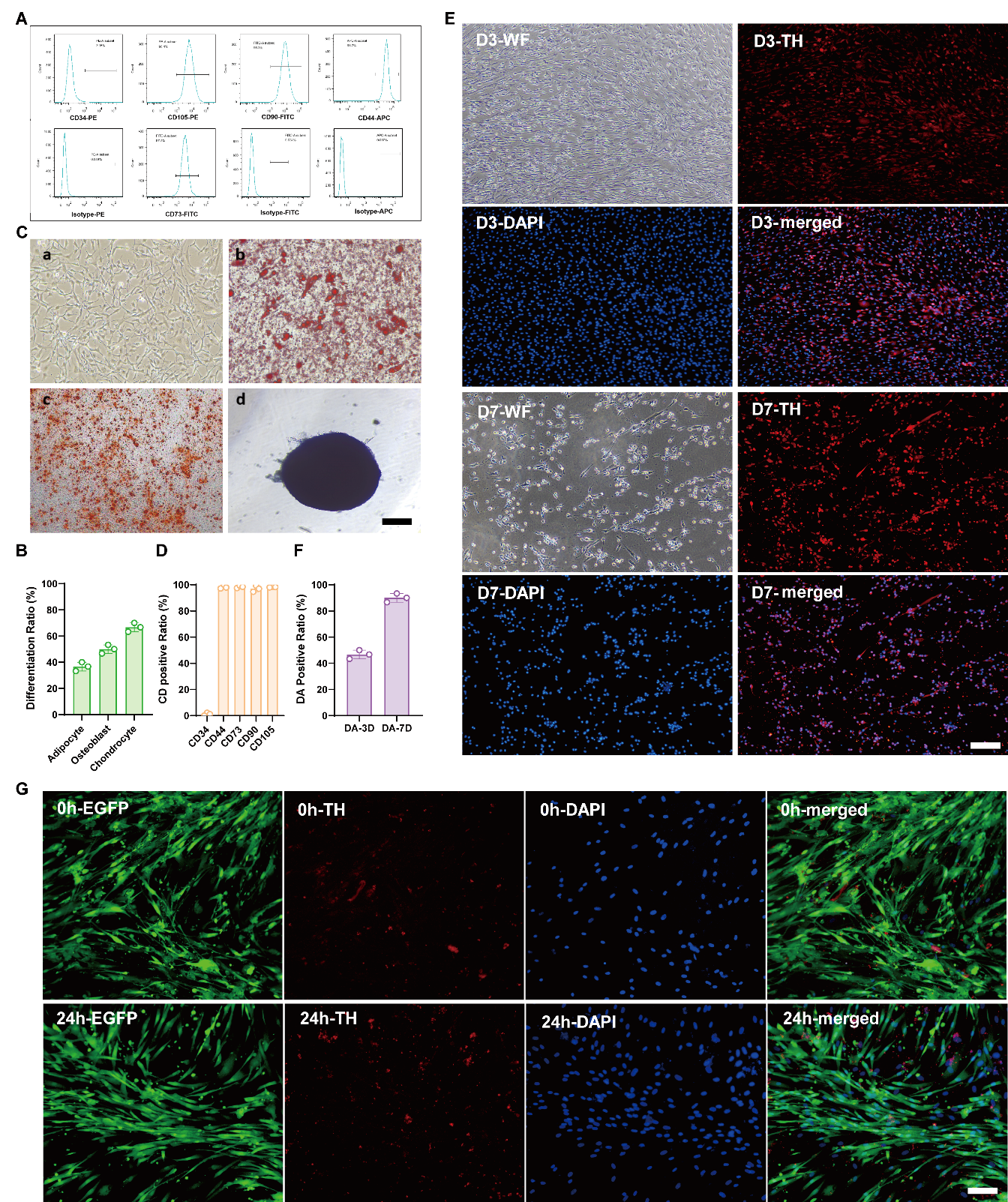


**Figure S1: Characterization of hADSCs and their differentiation into dopaminergic neuron like cells.** A, shows hADSCs positively express mesenchymal stem cell CD markers of CD44, CD73, CD 90 and CD105 (>97%). B, shows hADSCs could be conditionally induced into trileage mesodermal cells of adipocyte, osteoblast and chondrocyte. C, shows hADSCs could be gradually induced into dopaminergic neuron like (TH positive) cells. D, shows the statistic analysis for their trileage differentiation ratio, CD marker positive ratio and TH positive ratio at day 3 and 7 respectively. Scale bar=100 um, n=5. E, Characterization of EGFP labeled hADSCs by lentivirus and their differentiation into dopaminergic neuron like cells at 24 h before transplantation. Scale bar=100 um.


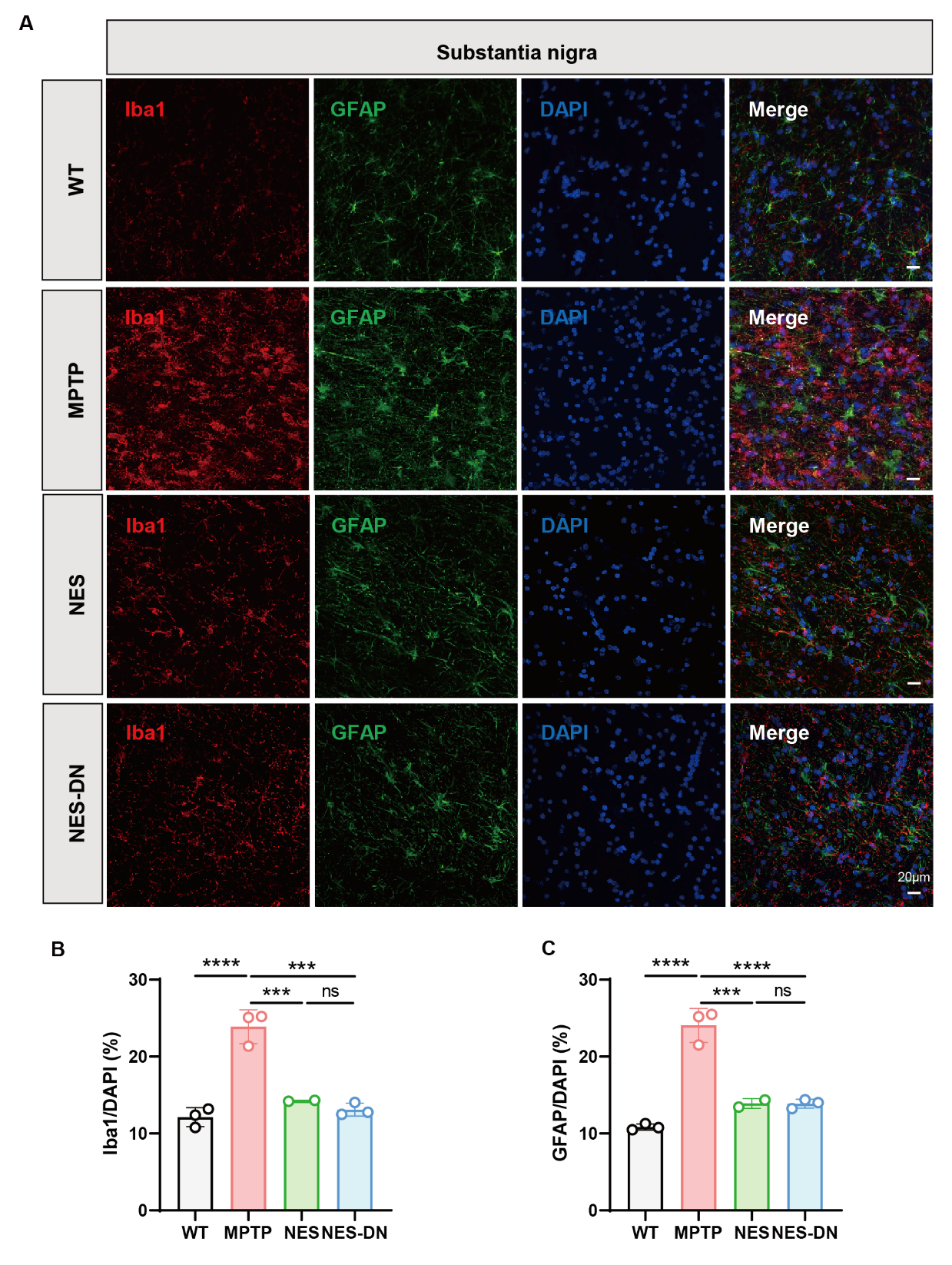


**Figure S2: NES and NES-DN treatments regulated the level of neuroinflammation in substantia nigra.** A, Representative images of Iba1 and GFAP immunofluorescence staining in substantia nigra, scale bar=20 μm. B and C, Quantitative statistics of Iba1 (B) and GFAP (C) positive cells. WT (n=3), MPTP (n=3), NES (n=2), and NES-DN (n=3), *** p<0.005, **** p<0.001.


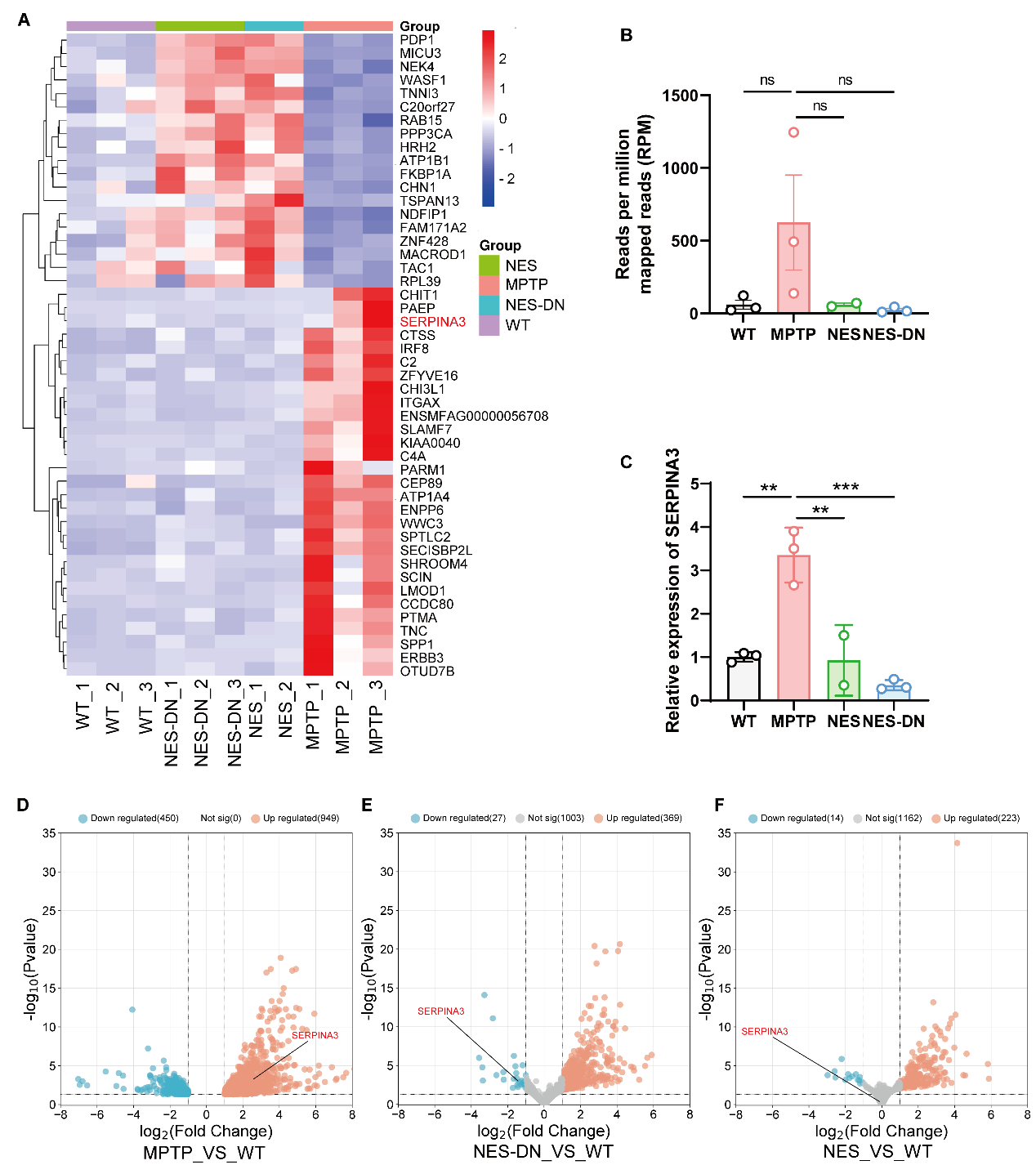


**Figure S3: Changes in SERPINA3 after NES and NES-DN treatment.** A, The common intersection gene heatmap of MPTP_VS-WT, NES-VD_MPTP, and NES-DN_VS-MPTP. B, Statistical chart of RNA level changes in SERPINA3. C, QPCR results display SERPINA3 RNA quantification statistical chart. WT (n=3), MPTP (n=3), NES (n=2), and NES-DN (n=3), ** p<0.01, *** p<0.005. D-F, Volcano map of MPTP_ VS_ WT (D), NES-DN_ VS_ WT (E) and NES_ VS_ WT (F).


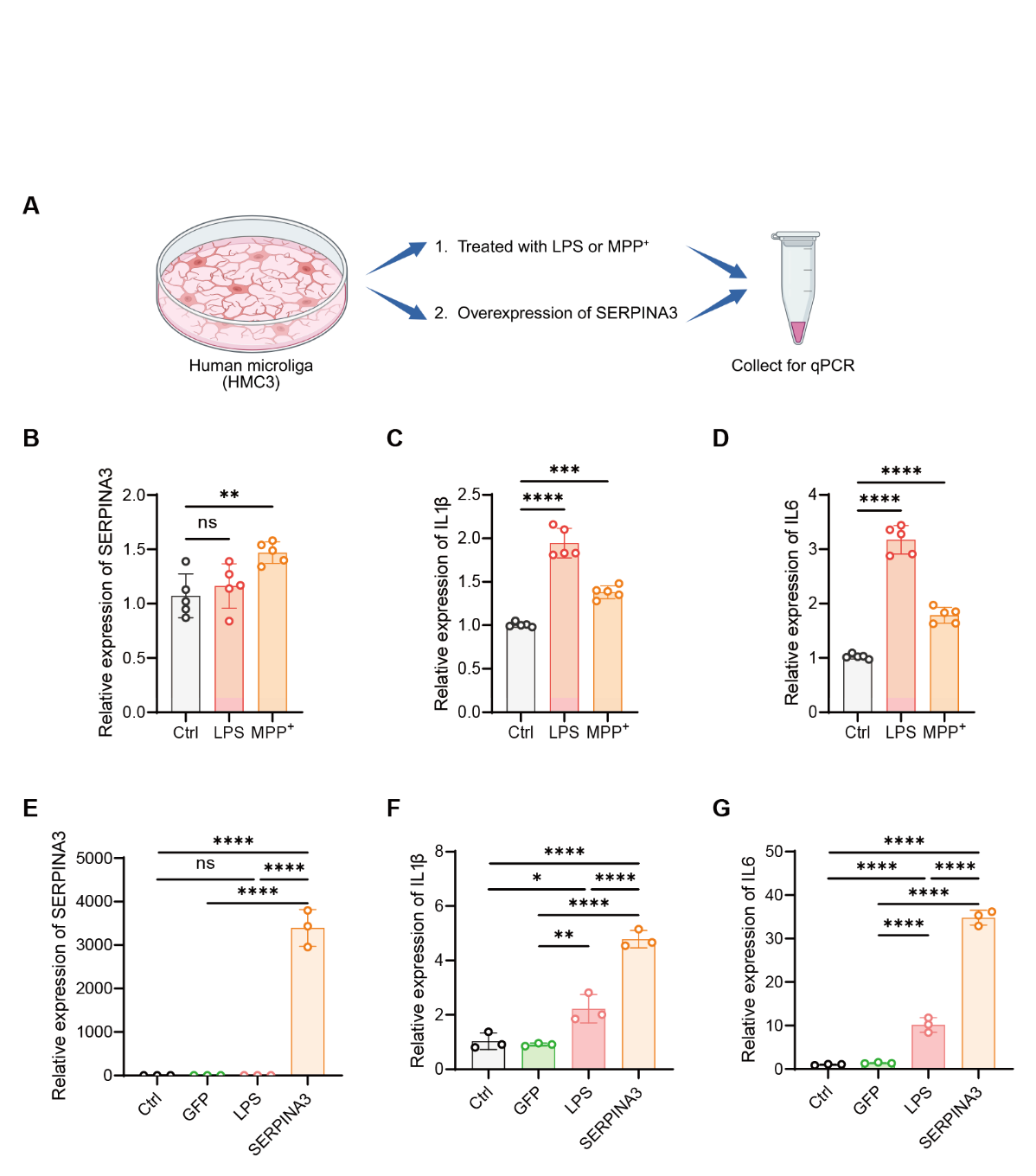


**Figure S4: MPP^+^ treatment or overexpression of SERPINA3 can induce an inflammatory phenotype in HMC3.** A. Schematic diagram of MPP^+^ processing or overexpression of SERPINA3 in HMC3. B-D. Expression levels of SERPINA3 (B), IL-1β (C), and IL-6 (D) after MPP^+^ and LPS treatment. Ctrl (n=5), MPP^+^ (n=5) and LPS (n=5), ** p<0.01, *** p<0.005, **** p<0.001. E-G. Overexpression of SERPINA3 leads to increased expression levels of SERPINA3 (E), IL-1β (F), and IL-6 (G). Ctrl (n=3), GFP (n=3), LPS (n=3) and SERPINA3 (n=3), * p<0.05, ** p<0.01, *** p<0.005, **** p<0.001.
